# A review on techno-economic assessment of *Spirulina* for sustainable nutraceutical, medicinal, environmental, and bioenergy applications

**DOI:** 10.1186/s40643-025-00888-3

**Published:** 2025-06-02

**Authors:** Musa Nasiru Musa, Ghazali Musa Jirgi, Zakariyya Uba Zango, Mannawi Nasiru Isa, Muhammad Abdurrazak, Adamu Ahmad Adamu, Ismael A. Wadi, Adekunle Akanni Adeleke, Zaharaddeen N. Garba, Usman Bello, Haruna Adamu, Ahmad Hosseini-Bandegharaei, Dmitry Olegovich Bokov

**Affiliations:** 1 Department of Biochemistry, College of Natural and Applied Science, Al-Qalam University Katsina, Katsina, 2137 Nigeria; 2 Institute of Semi-Arid Zone Studies, Al-Qalam University Katsina, Katsina, 2137 Nigeria; 3 Department of Chemistry, College of Natural and Applied Science, Al-Qalam University Katsina, Katsina, 2137 Nigeria; 4 Department of Physics, College of Natural and Applied Science, Al-Qalam University Katsina, Katsina, 2137 Nigeria; 5 Department of Biochemistry, School of Science and Information Technology, Skyline University Nigeria, Zaria Road Kano, Kano, Nigeria; 6 Department of Physiotherapy, Aminu Kano Teaching Hospital, PMB 3452, Kano, Nigeria; 7 Prince Sattam Bin Abdulaziz University, Basic Science Unit, Alkharj 16278, Alkharj, Saudi Arabia; 8 Department of Mechanical Engineering, Nile University of Nigeria, Abuja, Nigeria; 9 Department of Chemistry, Ahmadu Bello University, Zaria, 810107 Nigeria; 10 Biofuel and Biochemical Research Group, Department of Chemical Engineering, Universiti Teknologi, PETRONAS, 32610 Seri Iskandar, Malaysia; 11 Department of Chemistry, Abubakar Tafawa Balewa University, Gubi Campus, Bauchi, 740102 Nigeria; 12 Department of Environmental Management Technology, Abubakar Tafawa Balewa University, Yelwa Campus, Bauchi, 740272 Nigeria; 13 Faculty of Chemistry, Semnan University, Semnan, Iran; 14 Research and Innovation Cell, Rayat Bahra University, Mohali, Punjab India; 15 Department of Sustainable Engineering, Saveetha School of Engineering, SIMATS, Chennai, Tamil Nadu 602105 India; 16 Institute of Pharmacy Named After A.P. Nelyubin, Sechenov First Moscow State Medical University, 8 Trubetskaya St., Bldg. 2, Moscow, 119991 Russian Federation; 17 Laboratory of Food Chemistry, Federal Research Center of Nutrition, Biotechnology and Food Safety, 2/14 Ustyinsky Pr., Moscow, 109240 Russian Federation

**Keywords:** Antioxidant, Biodiesel, Environmental remediation, Nutraceutical, *Spirulina*

## Abstract

Global population growth underlies the need to explore alternative materials to address pressing challenges in food security, medicine, energy, and environmental pollution. *Spirulina* is a nutrient dense cyanobacteria that offers promising solutions to the aforementioned challenges, mainly due to its rich composition of proteins, vitamins, minerals, and bioactive compounds such as β-carotene and phycocyanin. These compounds confer various health benefits, including antioxidant, anticancer, anti-diabetic, antimicrobial, and anti-inflammatory properties, which make *Spirulina* a valuable dietary and therapeutic supplement. Essential fatty acids and its rapid growth rate also makes *Spirulina* a potential source of biodiesel for energy related applications. Additionally, *Spirulina*'s high porosity and variable functional groups endow it with remarkable biosorption properties for soil and wastewater remediation applications. The chemical structure and unique properties of *Spirulina* have been utilized to produce biotemplates for nanomaterials as well as the fabrication of functional composites for various applications. Thus, in this review, we have highlighted the broad potentials of *Spirulina* in diverse applications, emphasizing its eco-friendliness, economic viability, challenges, and the prospects of its biomass for sustainable, nutraceutical, therapeutic, energy related, and environmental applications.

**Graphical Abstract:**

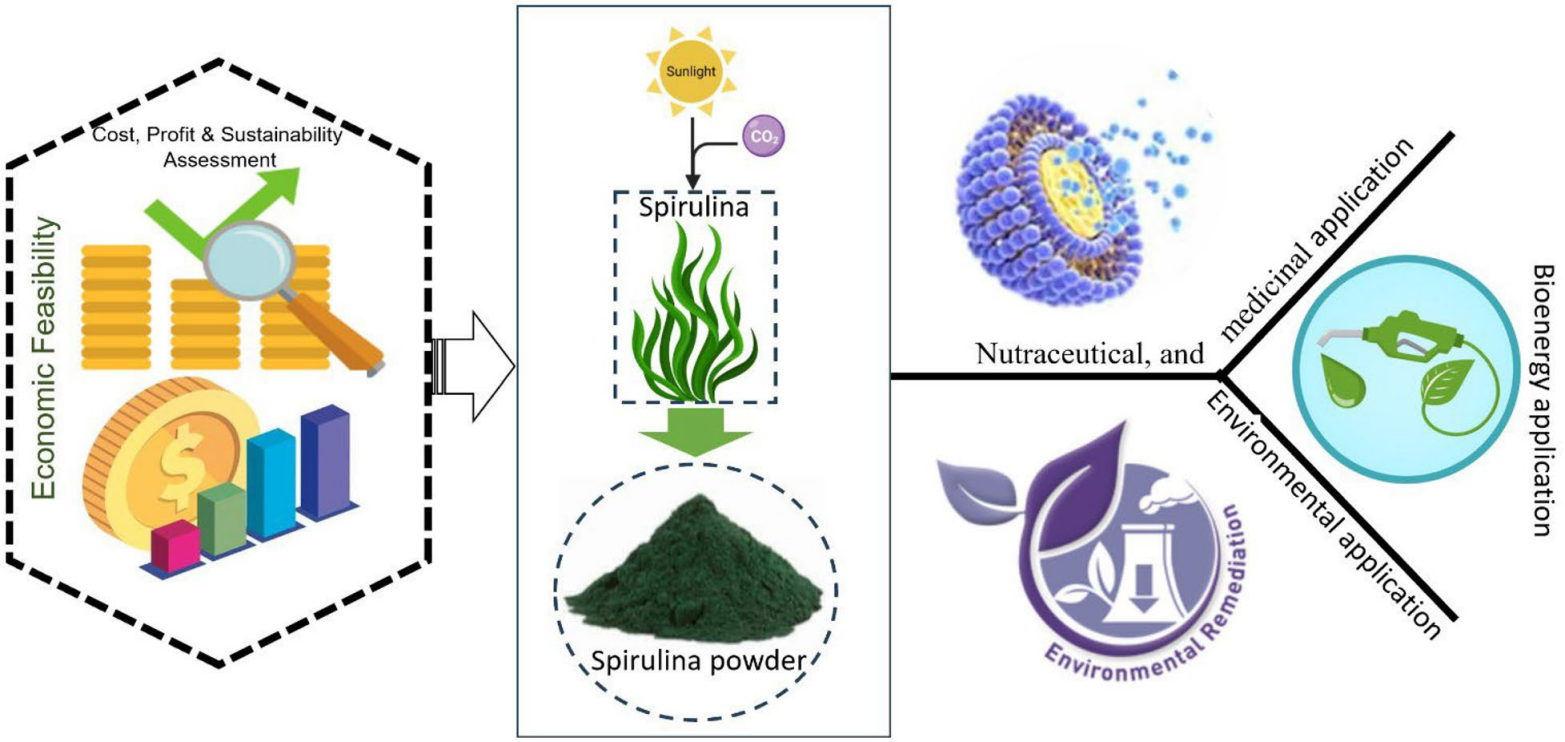

## Introduction

*Arthrospira*, commonly known as *Spirulina*, is an algal cyanobacterium which belongs to the *Oscillatoriaceae* family with characteristic filamentous helical structure (Michael et al. 2019). The two commercially available *Spirulina* species are *Arthrospira platensis* and *Arthrospira maxima*. They are widely used as food additives and dietary supplements (Ma et al. 2019). They are rich in protein (50–70%), essential fatty acids, vitamins, minerals, and amino acids with compositions significantly higher than most conventional food sources. Due to its lack of known toxicological effects, *Spirulina* has been extensively cultivated in Asia and America (Ragaza et al. 2020; Wuang et al. 2016). Additionally, *Spirulina* nutrients are easily preserved and stored without extensive processing (Usharani et al. 2015).

The primary interest in *Spirulina* for medicinal applications lies in its potential as a special food against various ailments (Wang et al. 2023). *Spirulina* is a one-stop solution for both medical and nutritional demands. Numerous medicinal and disease-preventing nutrients are contained in the *Spirulina*, including minerals, γ-linolenic acid, vitamin E, trace elements, B-complex vitamins, and other unknown bioactive compounds. It is particularly notable for its antioxidant and carotenoid compounds such as β-carotene, phycocyanin, zeaxanthin, cryptoxanthin, lutein, and superoxide dismutase (SOD) (Ma et al. 2019). *Spirulina* has been widely studied for activity against bacteria and viruses such as *S. Aureus*, *E. Coli*, influenza, measles, Herpes simplex virus (HSV-1), and human cytomegalovirus (Usharani et al. 2015). *Spirulina* can stimulate macrophage activity, enhance natural killer (NK) cell function, activate T-cells, and strengthen the immune response against infections (Calella et al. 2022).

*Spirulina* exhibits adsorption and bioaccumulation capacity, making it effective for bioremediation of heavy metals and wastewater from municipal waste, as well as the fish and dairy industries (Zhang et al. 2020). *Spirulina* favorably accumulates and tolerates zinc (Zn) and lead (Pb) but is highly sensitive to nickel (Ni) (Diaconu et al. 2023). Ni ions exert high toxicity to *Spirulina* by associating with metabolic and photosynthetic enzymes in addition to oxidative stress. *Spirulina* can also adsorb other metals, including cadmium (Cd), iron (Fe), and copper (Cu). Hence, the concentrations and type of metal are the main determinants for its application in the bioremediation of heavy metals (Diaconu et al. 2023; Zeraatkar et al. 2016).

### Cultivation and commercial production of *Spirulina*

Commercial *Spirulina* farming began in the 1970s in Mexico, and today, the Asia–Pacific region has witnessed a significant rise in *Spirulina* production (Ma et al. 2019). The International Energy Agency (IEA) estimates the annual production of *Spirulina* to be roughly 10,000 tonnes of dry biomass, with China accounting for half of this production (Costa et al. 2019a). For commercial cultivation, *Spirulina* is commonly grown using raceway pond systems (Kavitha et al. 2021). *Spirulina* can also be cultivated in laboratory and outdoor environments. Naturally occurring *Spirulina* can be cultivated and processed for commercialization by methods such as filtration, homogenization, pasteurization, and spray drying (Vellaiyan 2024). This has been practiced in the valley of Mexico by Sosa Texcoco Ltd, where it is collected at 2,200 m above sea level from the Lake Texcoco (Nawal K.Z. AlFadhly et al. 2022a, b).

Erlenmeyer flasks, reactors (photobioreactor), and L aquaria, collectively categorized as closed systems, can be used to grow the *Spirulina* in the laboratory, although hardly useful for commercial purposes (Costa et al. 2019a). Due to its requirement for tropical weather, laboratory production must adhere to precise conditions for water quality, macronutrients, micronutrients, light (light–dark cycle 12/12), temperature (30 °C), and pH between 8.5 and 10.5 (AlFadhly et al. 2022a, b). Closed systems have better control of growth conditions and higher biomass, coupled with reduced microbial contamination, loss of CO_2_, and evaporation. However, because they cost to construct and operate, their upscale has not been achieved (Costa et al. 2019a).

For commercial production, raceway ponds, which are open systems, are typically used all over the world to cultivate the *Spirulina*. Open systems cost less to operate, they can be easily maintained, are directly exposed to sunlight, accumulate less dissolved oxygen. Pond production follows four stages, which include culturing, harvesting, drying, and packaging, all of which are diligently monitored for optimal quality. Loss of biomass can be minimized during production by using a closed loop that recycles materials continuously. In a semicontinuous culture method, the biomass growth is collected from each pond after 24 h (Costa et al. 2019a). A pump with PVC pipes expels the culture into the facility, where it flows through screens for the *Spirulina* rinsing and concentration. In the filtration stage, it is pushed into a spray dryer to remove moisture and create a fine powder, and then it is returned to the pond (Michael et al. 2019). Conventional drying methods can significantly reduce the amounts of nutritional and bioactive compounds in the *Spirulina* (Ma et al. 2019). However, powdered and tablet supplements remain the most popular products of *Spirulina*.

Nutrients constitute a major factor in the production of the *Spirulina* and about 25% of the total production cost (Costa et al. 2019a). Zarrouk medium is the primary medium used in production systems but comes at high costs. Hence, attempts were made to develop affordable culture mediums that produce high-quality *Spirulina a* biomass equivalent to Zarrouk medium (AlFadhly et al. 2022a, b). Fertilizers and other affordable chemicals have been used to successfully replace the compounds in Zarrouk medium, in which ammonium nitrate proved to be a highly effective nitrogen source (Kumaresan et al. 2020). Some known media also include CFTR, OFERR, and Rao’s medium (Costa et al. 2019a). Wastewater from hatcheries and treated seawater can also support the growth of the *Spirulina* with lower efficiency than modified medium (Sandeep et al. 2015). Table 1 contained information on commercial production of *Spirulina* including some major suppliers, cultivation methods and the product type.

**Table 1:** Some commercial producers of *Spirulina*

Company	Country	Cultivation method	Capacity (tonnes/year)	Product type
Earthrise farms	USA	Semi-continuous/Open raceway pond	550	Powder and tablet food supplements
Cyanotech	USA	Semi-continuous/Open raceway pond	500	Powder food colorant
CHINA C.B.N	China	Semi-continuous/Open raceway pond system	1200	Powder and tablet food supplement/animal feed
DIC Corporation	Japan	Semi-continuous/Open raceway pond system	350	Powder and tablet food supplement/animal feed
Shuangfengbao Green *Spirulina* Co., Ltd	China	Greenhouse raceway pond	132	Powder food supplement/animal feed
Erdos Jiali *Spirulina* Co., Ltd	China	Greenhouse raceway pond	83	Powder and tablet food supplements
Luweibao *Spirulina* Bioeng. Company Ltd	China	Greenhouse raceway pond	58	Powder and tablet food supplements
Parry Nutraceuticals	India	Semi-continuous/Open raceway pond system	24	Powder and coated tablet
Fuqing King Dnarmsa *Spirulina* company Ltd	China	Semi-continuous/Open raceway pond system	1600	Powder and tablet food supplements

The information is obtained from (Costa et al. 2019b; Lafarga et al. 2021; Lestingi et al. 2024; Lu et al. 2011; Villaró-Cos et al. 2024)

## Biochemical composition

The major constituents of *Spirulina* are proteins, carbohydrates, and lipids, which are produced by photosynthesis. *Spirulina* is particularly rich in protein, which can make up over 60% of its dry matter with an extensive proportion of amino acids (Lestingi et al. 2024). The carbohydrate composition of *Spirulina* is estimated at 15–20% of its dry matter, mainly as polysaccharides. *Spirulina* also has lipid composition between 6.4 and 14.3%, depending on the growth condition (Fattah et al. 2020; Neag et al. 2022). The lipids composition mainly include polyunsaturated fatty acids (PUFA) such as palmitic acid, γ-linolenic acid (GLA), eicosapentaenoic acid (EPA) and docosahexaenoic acid (DHA) (Spínola et al. 2024a). *Spirulina* is a valuable source of minerals, including calcium, magnesium, potassium, zinc, iron, and selenium among others (Carcea et al. 2015). It also contains vitamins, namely Vitamins B (B1, B2, B3, B6, B9, and B12) and vitamin K (Spínola et al. 2024a). Furthermore, *Spirulina* contains significant levels of bioactive compounds including carotenoid pigments (β-carotene), chlorophylls, and phycobiliprotein complexes such as C-phycocyanin and allophycocyanin. Tocopherol, flavonoids, and phenolic such as salicylic, trans-cinnamic, synaptic, chlorogenic, quinin, and caffeic acids are also present among others (Lestingi et al. 2024).

Table 2 presented information about the biochemical compositions of the *Spirulina* as reported in some literatures.

**Table 2:** Biochemical composition of *Spirulina*

Compound	Unit	Composition
Protein	%	50–70
Carbohydrate	%	15–20
Lipids	%	6.4–14.3
Saturated fatty acids	%TFA	49.2
PUFA	%TFA	41.9
Monounsaturated fatty acids	%TFA	8.9
n-6 fatty acids	%TFA	40.4
n-3 fatty acids	%TFA	0.4
γ-linoleic acid	%TFA	12.9–40.1
Palmitic acid	%TFA	25.8–47.6
Eicosapentaenoic acid	g/kg	< 2.5
Docosahexaenoic acid	g/kg	< 3.0
Sodium	g/kg	4.5–96.2
Potassium	g/kg	6.4–29.1
Phosphorous	g/kg	1.2–22
Magnesium	g/kg	0.77–4.0
Calcium	g/kg	0.23–14.0
Iron	mg/kg	106–1800
Manganese	mg/kg	13–550
Zinc	mg/kg	0.4–40
Copper	mg/kg	0.4 -18.7
Vitamin B1	mg/kg	5–50
Vitamin B2	mg/kg	30–46
Vitamin B3	mg/kg	130–150
Vitamin B6	mg/kg	4–50
Vitamin B9	mg/kg	0.3–99.2
Vitamin B12	mg/kg	0.06–3.1
Vitamin K	mg/kg	22
Vitamin E	mg/kg	24.6–750
Total carotenoids	g/kg	0.3–26
β-carotene	g/kg	0.02–2.3
Total chlorophylls	g/kg	1.2
C-phycocyanin	g/kg	94.9–251
Allophycocyanin	g/kg	23
Total phenols	g/kg	2–17.3
Total flavonoids	g/kg	1–9

(Fattah et al. 2020; Neag et al. 2022; Lestingi et al. 2024; Spínola et al. 2024a, b), *TFA* Total fatty acid

### Nutraceutical properties of *Spirulina*

Global population growth is accompanied by an increasing food demand to supplement the rise in population. By the year 2050, the world population will eventually reach 10 billion, further necessitating the need to produce more food to meet an ever increasing demand (Bumandalai et al. 2024). Thus, microalgal-based diets are considered as good alternatives to complement plant and meat-based diets because of their sustainability, positive impact on public health, environmental conservation, and food security. The prospects of the *Spirulina*-based food are credited to its rich nutritional composition (Luo et al. 2024).

*Spirulina*’s protein composition is comparably higher than meat (43%), eggs (12.6%), soy (37%), whole milk powder (26%), and yeast (39%) (Bertsch et al. 2021; Thevarajah et al. 2022). It is an excellent protein source that can also benefit both vegetarians and vegans. It has sufficient composition of all essential amino acids that meet standard dietary recommendations (Thevarajah et al. 2024). The World Health Organization (WHO) and the US Food and Drug Administration (FDA) have approved *Spirulina* as a safe and relevant superfood, with NASA also recognizing its potential use for space missions (Fais et al. 2022; Sorrenti et al. 2021). *Spirulina* is also a source of other essential nutrients that are necessary to maintain a healthy diet.

### Medicinal properties of *Spirulina*

*Spirulina*'s rich nutritional makeup can provide several health benefits such as Immunomodulation, antioxidant, antiviral, anticancer, and antibacterial properties (Chwil et al. 2024; Mirza et al. 2025). It can also be beneficial in preventing anaemia, hyperlipidaemia, obesity, diabetes, heavy metal and chemical toxicity, inflammatory allergic reactions, radiation damage, and malnutrition (Sahil et al. 2024; Shah et al. 2024a). *Spirulina*’s medicinal activities are often presumed to be based on species. For instance, the immune system is enhanced by *S. platensis* in both humans and animals. Administering *Spirulina* enhances immune activity in patients with cancer, AIDS, and other viral diseases (Akbarizare et al. 2020; Prabakaran et al. 2020). *Spirulina* boosts the immune system due to increased production of antibodies and the activity of immune cells (Prabakaran et al. 2020). Consumption of *Spirulina* supplements has been strongly recommended for patients with weak immune system, due to its immune system boosting and detoxification properties (Wells et al. 2017).

**Fig. 1:**
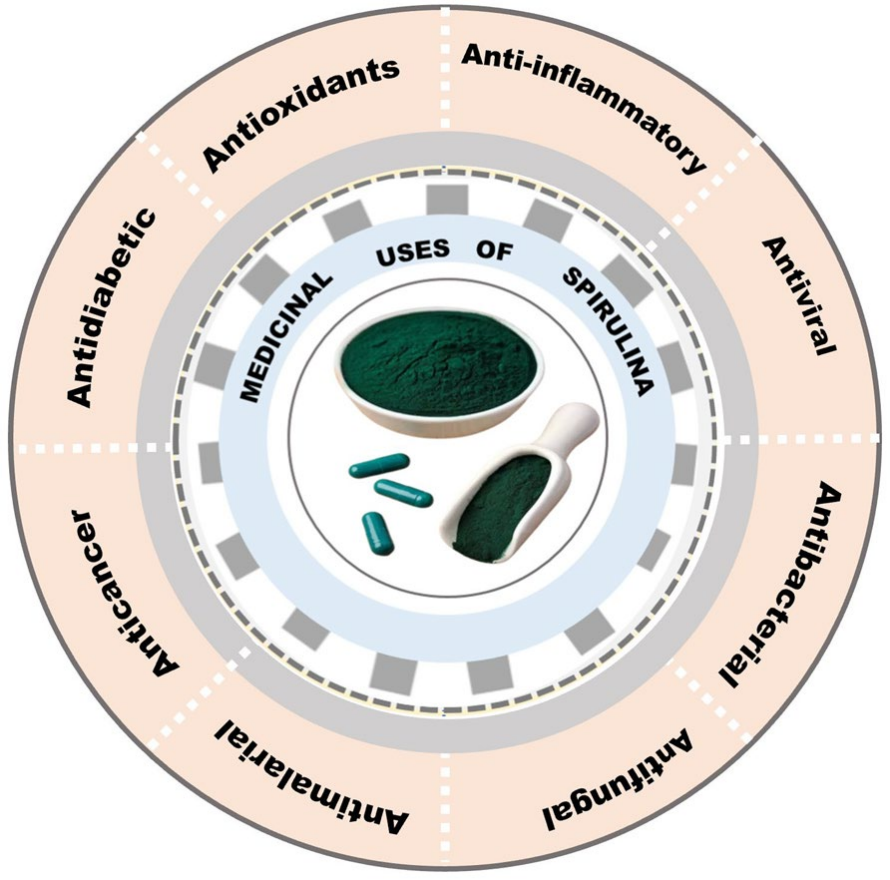
Some bioactive properties of *Spirulina*

The blue pigment in *Spirulina*, phycocyanin, possess potent antioxidant and anti-inflammatory properties, which are suitable for combating oxidative stress in the body. The anticancer properties of *Spirulina* also result from its antioxidant and antimutagenic activities (Maddiboyina et al. 2023; Tajvidi et al. 2021). It has antibacterial properties and can be used to fight off bacterial infections (Ghallab et al. 2024). Some studies suggest *Spirulina* can help lower blood pressure, contributing to cardiovascular, kidney and liver health (Bin-Jumah et al. 2021). Figure 1**,** highlights some of the bioactive properties of *Spirulina* and expatiated in the sections below.

#### Antimicrobial activity of *Spirulina*

*Spirulina* is known to have antimicrobial properties against the growth of microorganisms such as bacteria, viruses, and fungi. *Spirulina*'s antimicrobial properties are attributed to bioactive compounds, fatty acids, and polysaccharides in its products and extracts (Singh et al. 2021; Alshuniaber et al. 2021). The antiviral properties of *Spirulina* and its products have been well studied, especially against enveloped viruses including influenza, HSV, and HIV. By preventing viruses from entering host cells, Ca-SP, a sulphated polysaccharide isolated from *Spirulina* has been demonstrated to cause suppression in viral replication.

Phenols and peptides in *Spirulina* can also inhibit the growth of pathogenic bacteria (Spínola et al. 2024a). Peptides can interfere with microbial growth and complement the body’s defense mechanism against infections (Nabti et al. 2023). *Spirulina*’s antimicrobial property can also be attributed to the combined action of compounds such as γ-linolenic acid, active fatty acids, lauric and palmitoleic acids. A possible explanation is that lipids disrupt the membranes of microbial cells in yeasts, fungus, and bacteria, which result in their death (Bellahcen et al. 2020; Ilieva et al. 2024). Additionally, they can reach the bacterial membrane and cause its disintegration by penetrating its cell wall's dense peptidoglycan meshwork without causing any noticeable alteration (Ilieva et al. 2024). Gram-positive bacteria are more sensitive than gram-negative bacteria because they contain a single peptidoglycan layer (Tavares et al. 2020).

#### Antioxidant activity of *Spirulina*

Pigments such as chlorophyll, phycocyanin, β-carotene, and phycoerythrin are natural antioxidants that neutralize free radicals, which give *Spirulina* its antioxidant properties (İlter et al. 2018). According to a study, *Spirulina* contains carotenoids, phenolics, and tocopherols, which also have demonstrated antioxidant activity (Han et al. 2021). Organic acids such as salicylic and trans-cinnamic acids from phenolic compounds of *Spirulina* exerted antioxidant effects both in combination and independently (Han et al. 2021).

*Spirulina* can increase reduced glutathione levels in cells, while maintaining the activities of glutathione reductase (GR), glutathione peroxidase (GPx), and selenium-dependent glutathione peroxidase (GPx-Se) vitamins (Kumar et al. 2022a, b). Aqueous and ethanolic extracts of *Spirulina* both contain significant amounts of Catalase (CAT), Superoxide Dismutase (SOD), and antioxidant vitamins (C and E). However, aqueous extracts showed higher activities for both the enzymes and vitamins (Kumar et al. 2022a, b). Oxidative stress is counteracted by both enzymatic and lipophilic antioxidants (Kumar et al. 2022a, b).

#### Effects against hyperlipidaemia

*Spirulina*’s efficacy has been studied against lead acetate-induced hyperlipidaemia and oxidative stress (Gargouri et al. 2020). Exposure to lead acetate causes an increase in peroxidation, tissue damage due to oxidative stress, and hyperlipidaemia (Gargouri et al. 2020). *S. maxima* was an effective antioxidant for neutralizing free radicals and effective in reducing oxidation of lipids (Gargouri et al. 2020). It prevents notable alterations in liver and plasma lipid levels and maintains antioxidant status of the liver and kidney. Oral administration of *Spirulina* was demonstrated to decrease blood pressure and lipid concentration, particularly low density lipoprotein cholesterol (LDL-C) and triacylglycerols. These effects are mostly ascribed to GLA and phycocyanin, which control lipid metabolism and keep fat from building up in blood vessels (Torres-Duran et al. 2007). It also indirectly altered the levels of lipoprotein cholesterol and total cholesterol. Water *Spirulina* extract can prevent dietary fat from being absorbed through the digestive tract by inhibiting pancreatic lipase (Gargouri et al. 2020; Saraswathi and Kavitha 2023).

#### Effects against diabetes, obesity, and hypertension

*Spirulina* is widely used because of its potential therapeutic effects for the management of various health conditions, including diabetes, obesity, and hypertension. It possess hypoglycaemic effects which helps in reducing the levels of sugar in the blood. The *Spirulina* as a supplement helps in improving the sensitivity of insulin and controls blood glucose in diabetic patients (Hu et al. 2019; Krishnan et al. 2024). The presence of bioactive compounds contribute effectively to its antidiabetic efficacy by reducing and regulating the metabolism of glucose and insulin (Prabakaran et al. 2020). It helps in prevention of obesity and weight management. Its high content of fiber and protein also help in appetite reduction, improves satisfaction, and aid in the metabolism of fats. Additionally, the antioxidant and anti-inflammatory effects of *Spirulina* is important for obesity control and other complications caused by metabolic disorders (Krishnan et al. 2024; Moradi et al. 2019). The ability of *Spirulina* to reduce oxidative stress is paramount to improving endothelial function and modulation of blood pressure among individuals with hypertension complications (Münzel and Daiber 2019). *Spirulina* has been useful for lowering blood pressure and risks from cardiovascular complications (Münzel and Daiber 2019).

According to a study, consumption of *Spirulina* causes suppression of glucose level (Liu et al. 2022). The water-insoluble part of has been responsible for the suppression. Similar results were seen in another research. For example, in a clinical investigation on diabetic patients, fasting blood sugar levels were found to drastically decrease after 21 days of taking 2 g of *Spirulina* daily. Body weight reduction in obese patients have been observed following intake of *Spirulina* 3 times per day for a period of 4 weeks. Suppression of high blood pressure in rats has been linked with *Spirulina* (Moradi et al. 2019). A vasodilating activity by *Spirulina* on the aortic rings in rat probably discovered for cyclooxygenase-dependent products (De Freitas Brito et al. 2019). According to investigation, impact of polysaccharides and phycocyanin on the hematopoietic system of mouse bone marrow and peripheral blood has been reported (De Freitas Brito et al. 2019). A clinical study conducted has shown the effect of *Spirulina* for the decrease in the damage of endothelia for the patient with systematic arterial hypertension. In another study, a reduction in oxidative stress was reported after administering the *Spirulina* to the patients (Martínez-Sámano et al. 2018).

#### Effects against nephrotoxicity

The *Spirulina* potentiality against nephrotoxicity have been studied. This refers to a condition where kidney is damage because of exposure to toxic substances over long time. Researchers have explored the *Spirulina* efficiency for renal infections (Bin-Jumah et al. 2021; Dhamak and Amrutkar 2023). The major key for nephrotoxicity is oxidative stress due to tissue inflammation. The *Spirulina* has potentiality of protecting renal cells from injury due to its scavenging free radicals (Sayed et al. 2020). Chronic inflammation has been the major indicator of nephrotoxicity. The *Spirulina* possessed anti-inflammatory effects via the inhibition of the cytokines which caused the inflammation and injury of the kidney (Sayed et al. 2020). The immune response of the immune can be modulated by the reduction of the inflammation of the kidney.

Also, the polysaccharides constituents in the *Spirulina* potentially bind with heavy metals and toxins in the kidney which facilitates their faster excretion from the body. Thus, the effect due to the detoxification is important for the toxic substances accumulation in the kidneys and possible ejection from the body (Sayed et al. 2020). One study revealed that the *Spirulina* as supplement is effective in the promotion of blood flow which facilitates the function of kidney and cause reduction in the glomerular damage, which are vital for the cells regeneration in the kidney (Aziz et al. 2018). Thus, the *Spirulina* is important as supplement for improving the renal function, and alleviation of nephrotoxicity complications.

##### Anti-inflammatory effects

The *Spirulina* is well known for its potentiality against inflammation. The bioactive substances like the polysaccharides, phycocyanin, phycocyanobilin, are potent for managing various conditions of inflammation. The bioactive compounds fabricate pro-inflammatory cytokines, like the interleukin-6 (IL-6) and tumour necrosis factor-alpha (TNF-alpha) which are active against the body inflammation (Wu et al. 2016). The *Spirulina* helps in alleviating the body inflammatory response by lowering the release of the mediators. It also helps in modulating the immune response by regulating the activities of the immune cells, including T cells, B cells, and macrophages (Ghamry et al. 2023). The effect of the modulation of immune is effective in the maintenance of its balance and prevention of excessive inflammation caused by the chronic conditions (Abu-Taweel et al. 2019).

Thus, studies have the effect of the *Spirulina* for the alleviation of body inflammation. The free bilirubin important role as potent oxidase activity inhibitor (Abu-Taweel et al. 2019). It has also been discovered that the chromophore phycocyanobilin (PCB), which is abundant in cyanobacteria and blue-green *Spirulina* like *Spirulina*, severely inhibits this enzyme complex (Abu-Taweel et al. 2019). This may be because PCB is rapidly converted to phycocyanorubin in mammalian cells (Wu et al. 2016). Until commercially available synthetic PCB or PCB-enriched *Spirulina* extracts are available, consuming entire *Spirulina* is the most practical and least expensive approach to administer PCBs (Al-Qahtani and Binobead 2019). *Spirulina* has been shown to inhibit the enzyme cyclooxygenase-2 (COX-2). By blocking COX-2 activity, *Spirulina* can help reduce inflammation and pain associated with inflammatory disorders (Al-Qahtani and Binobead 2019). A positive impact on gut health has been recognized administering a *Spirulina*. It shows to promotes beneficial gut bacteria growth and maintain healthy gut microbiota composition (Calella et al. 2022). A balanced gut microbiome is essential for controlling inflammation and supporting overall immune function.

#### Anti-cancer and immune effects

Chemotherapy has been the most widely used technique for the treatment of cancer. Various medications are used to stop the spread of cancer cells (Tajvidi et al. 2021). These medications are associated with various toxicities, which make them unpleasant at best and potentially deadly at worst. The use of chemotherapeutic drugs results in number of side effects such as body fatigue, appetite loss, mouth sores diarrhoea nausea, and hair loss. Thus, novel medications are needed for the cancer treatments. *Spirulina* has demonstrated promising features according to preclinical studies prevention and treatments of cancer (Lu et al. 2023).

The *Spirulina* helps in activating the natural killer (NK) cells and when combined with the BCG-cell wall skeleton, it caused the development of adjuvant-based anticancer immunotherapy (Subramaiam et al. 2021). Studies conducted on the *Spirulina* anti-cancer efficiency include the model animals such as rats (Subramaiam et al. 2021). The *Spirulina* modulates the immune system and enhance cellular immunity as well as improve the activity of NK cells, which are vital for the surveillance of immune against the cancer cells (Lu et al. 2023). A study suggested that *Spirulina* induces apoptosis in cancer cells, causing the inhibition growth and death of the cancer cells (Subramaiam et al. 2021). The mechanism of the *Spirulina* action has been ascertained by collection of blood cells among volunteers who had consumed hot water containing *Spirulina* extract orally and had their immune levels assessed (Lu et al. 2023). Their immune system was found to improve upon the *Spirulina* consumption which was attributed to the consumption, Therefore, in humans, *Spirulina* directly affects myeloid lines and may potentially have a direct or indirect effect on (NK) cells (Lu et al. 2023).

#### Other health benefits

Consumption of *Spirulina* has numerous health benefits. For instance, *Spirulina* has been suggested to help detoxify the body by binding to heavy metals and toxins, aiding in their elimination. The *Spirulina* is generally considered safe for most people, including individuals with certain health conditions, allergies, or sensitivities (Trotta et al. 2022). It can be incorporated into diet and has vital health due to its rich nutrients content. The consumption of *Spirulina* among athletes helps in boosting their energy levels and increase their endurance.

### Side effects of Spirulina

Individual with certain allergies and sensitivities are advised to consult healthcare providers before adopting *Spirulina* in their diet to avoid side effects and complications. Gastric hyperacidity and poor digestion can cause upset stomach, hiccups, moderate diarrhoea, nausea, and constipation. Lethargy, persistent hunger, and dizziness are possible symptoms of anaemia and hypoglycaemia. As concentrated protein, *Spirulina* has the potential to increase body warmth. Excessive fat burning by the body may lead to sleep difficulties and excitation. In these circumstances, taking *Spirulina* exclusively in the morning is advised. Headaches are mostly just a very short-lived, sporadic healing crises, though they can also be caused by poor digestion (Wang et al. 2019).

Despite the non-toxic nature of the *Spirulina* to aquatic organisms, it possessed the tendency of heavy metals and other contaminants bioaccumulation from its environment (Essid et al. 2020; Guimarães et al. 2021). When harvested in polluted water area, it could pose risks to aquatic organisms and the ecosystems they inhabit. The presence of *Spirulina* at higher concentration in aquatic environment may indicate eutrophication, leading to degraded water quality, which can harm aquatic organisms through oxygen depletion or harmful algal blooms (Michalak et al. 2020). Also, large concentration of the *Spirulina* in aquaculture food without a balanced diet, might lead to nutritional imbalances or deficiencies in aquatic species (Shah et al. 2024b). If *Spirulina* is introduced into non-native environments, it could potentially disrupt local ecosystems by outcompeting native species (Michalak et al. 2020). Overall, while the *Spirulina* itself is non-toxic in nature, the environmental conditions upon which its cultivated may cause harmful effects to the body. As such, it is important to obtain the biomass from clean environment or source from trusted suppliers.

### Clinical trials

*Spirulina* has been the subject of various clinical investigations aiming to assess its therapeutic potential. Research indicates potential health benefits, including immune system support, anti-inflammatory effects, improved lipid profiles, and antioxidant properties. Studies have also investigated *Spirulina*’s potential role in managing conditions such as obesity, diabetes, cardiovascular diseases, allergies, and cancer (DiNicolantonio et al. 2020; Hernández-Lepe et al. 2019; Zeinalian et al. 2017). A randomized, double-blind, placebo-controlled study conducted has evaluated the effects of taking *Spirulina* supplements on immune response in healthy adult in Korea (Park et al. 2008). The findings indicated an improvement in the immune markers among the individuals who consumed the supplements. This includes an increase in the production of antibody as well as T-cell activity boosting which confirmed the enhancement in the functions of the body immune (Park et al. 2008).

Several studies have looked into the effects of *Spirulina* on lipid profiles, liver and cardiovascular problems (Gupta 2025; Pandey and Singh 2022). For the *Spirulina* effect on liver, clinical studies have been conducted to determine the hepatoprotective effects on patients with liver fibrosis and chronic liver disease. The individuals examined after the *Spirulina* consumption have experienced improvements in liver function tests, along with reduction in the liver inflammation markers, which suggest the effect of the *Spirulina* on improving the liver health (Mazloomi et al. 2022; Yousefi et al. 2019). According to a finding, consumption of *Spirulina* supplementation has significant effect on total cholesterols reduction. These include LDL cholesterol, and triglycerides while increasing HDL cholesterol which suggests its potential benefits for for individuals with cardiovascular problems (Prete et al. 2024). While some studies show promising results, further research is needed to fully understand its mechanisms of action, optimal dosage, and long-term effects on human health.

### Safety considerations

*Spirulina* is often produced in open ponds, which are vulnerable to contamination by various microorganisms, including some toxin-producing cyanobacteria. A study examined the microbial population of commercially available *Spirulina* products and detected several potentially pathogenic bacteria, including *Bacillus cereus* and *Klebsiella pneumoniae*. Microcystin toxins were detected in all the products at levels that could lead to consumers exceeding their recommended daily limits. This highlights microbiological safety issues associated with commercial *Spirulina* products (Rhoades et al. 2023). Although rare, there have been reports of allergic reactions to *Spirulina*, ranging from mild symptoms to severe anaphylaxis. A literature review identified five cases of *Spirulina* allergy, with four classified as anaphylaxis. This underscores the need for awareness regarding potential hypersensitivity in susceptible individuals (Gromek et al. 2024). Some *Spirulina* supplements have also been found to be contaminated with microcystins, albeit at levels below the limit set by the Oregon Health Department. Microcystins can cause gastrointestinal upset, such as diarrhoea, flatulence, headache, muscle pain, facial flushing, and sweating. Chronic exposure may lead to liver damage. The effects of chronic exposure to even low levels of microcystins are a concern due to the risk of toxicity to several organ systems. These toxic compounds are not produced by *Spirulina* itself but can occur if *Spirulina* batches are contaminated with other, toxin-producing, blue-green *Spirulina* (Rhoades et al. 2023).

### Regulatory challenges

Ensuring the purity and safety of *Spirulina* products is paramount. Contaminants such as heavy metals, pathogenic bacteria, and toxins can pose health risks. Regular monitoring and adherence to good manufacturing practices are essential to mitigating these risks. The lack of standardized production methods also leads to variability in the composition of *Spirulina* supplements. This inconsistency complicates the assessment of its efficacy and safety across different products. Another challenge is that in many countries, *Spirulina* is marketed as a dietary supplement, which may not require rigorous pre-market evaluation. This classification can result in products reaching consumers without comprehensive safety assessments. Consumers should therefore opt for products from reputable manufacturers that adhere to stringent quality control measures. Ongoing research and regulatory vigilance are also critical to ensuring safe consumption of *Spirulina*.

## Biosorption properties of *Spirulina*

The *Spirulina* biosorption features as well as that of other microalgal biomass been exploited for contaminants remediation in both soil and water environments (Muhamad et al. 2023; Rawindran et al. 2023). The capability of the *Spirulina* to adsorb contaminants from soil and wastewaters has rendered the biomass highly significant in environmental remediation. It possessed significant advantages because of its relative abundance, higher yield, and lower costs of production. It is potential for elimination of heavy metals and organic pollutants from soil and wastewater (Bonyadi et al. 2022; Rezaei 2016). The *Spirulina* possess wider surface area-to-volume ratio, which sufficiently provides active sites for the adsorption of the contaminants. Moreover, the various functional groups in the *Spirulina* including the amino acids, hydroxyl groups and carboxyl groups create abundant adsorption sites for uptake and interaction with the contaminants via chemical and physical processes (Cepoi et al. 2020; Diaconu et al. 2023).

The *Spirulina* served as low-cost option for physical and biological remediation compared to chemical methods. Several findings have been reported on the heavy metals removal from soil using the *Spirulina* (Diaconu et al. 2023; Moubayed and Al-houri 2022). Also, reports are available in the literature for the treatment of smelter and refinery effluents, dyes, pharmaceuticals, and wide spectrum of organic contaminants, focusing more on industrial wastewater treatment or, more broadly, bioremediation of aquatic systems (Alves et al. 2020; Choi et al. 2020). Reports on removal of heavy metals and inorganic contaminants, from the wastewater is also available (Palaniswamy and Veluchamy 2017). Its use in the soil and wastewater remediation contributes to environmental sustainability and serve as a valuable tool for addressing the persistent problem of the soils and waters contamination.

### Biosorption of heavy metals from soil

Heavy metals have been one of the major contaminants of soil in agricultural land. They resulted from various sources such as the over use of pesticides which contained heavy metals that potentially accumulate in the environment over time (Adeniyi et al. 2022; Isiyaka et al. 2021). Use of phosphate-based fertilizers which contain arsenic, cadmium, lead in trace amount, use of untreated sewage that contained the heavy metals as fertilizers, contaminated run-off from mining and smelting industries near agricultural farmlands, industrial wastes from manufacturing industries that leach into the soil, urban construction also introduce heavy metals to the soil, chemical spills, landfill leakage, recycling of materials, atmospheric deposition, weathering and erosion as well as other natural processes (Adamu et al. 2024a, b; Armaya et al. 2020). The heavy metals presence in the soil posed significant threat to the soil viability, agricultural productivity, soil microorganisms, animals, and human health (Armaya et al. 2020). Thus, the need to eliminate them from the soil.

The *Spirulina* biomass are used as potential adsorbent for heavy metals removal. Utilizing the algal biomass in contaminated soils efficiently helps in reducing the concentrations of heavy metal which in turn helps in the mitigation of their toxic impacts to plants and organisms (Nithya et al. 2019; Sayadi et al. 2019). The *Spirulina* biomass has been effective for biosorption of heavy metals, like chromium (Cr), nickel (Ni) copper (Cu), zinc (Zn), cadmium (Cd), mercury (Hg), and lead (Pb) from the soil environment (Anemana et al. 2022; Musio et al. 2022). According to a study, the cyanobacterium biomass remarkable efficiency for the biosorption of heavy metals in multi-component system at concentration range of 2.5–10 mg/L (Cepoi et al. 2020). The mode of the heavy metal biosorption has been dependent on *Spirulina* growth stage which shows higher uptake of Cr (IV) and Fe (II) compared to Cu, Ni, and Zn (Cepoi et al. 2020). The *Spirulina* methylation also helps in facilitation of the heavy metals biosorption which shows higher uptake of the contaminants (Aravind et al. 2023; Malletzidou et al. 2025). The number of carboxylate groups at the surface of the biomass was reduced upon the methylation, and new functional groups such as ether (-C-OCH_3_), amines (-NH_2_, -NHR), and "free" carboxy groups (-COOH) emerged which significantly enhanced the biosorption efficiency (Malletzidou et al. 2025). The Cd (II) biosorption onto *Spirulina* grown under static magnetic field (SMF) has been studied (Shao et al. 2018). Accordingly, the increase in the uptake of the heavy metal at the initial concentrations of the Cd (II) (10–15 mg/L) improved the biosorption efficiency from 61.5–79.7%. Interestingly, the biosorption was higher for the *Spirulina* grown under the SMF which has efficiency in the range of 82.3–91.4% (Shao et al. 2018).

The biosorption process is dependent on factors such as the contact time, concentration, temperature and pH as well as the competing ions in the soil. Also, it is described by different mechanisms such as bioaccumulation where the heavy metals get accumulated within the *Spirulina*, ion exchange where the heavy metals replaced the cations at the surface of the *Spirulina* biosorbent and, surface complexation which exists between the heavy metals and the functional moieties at the surface of the *Spirulina* (Finocchio et al. 2010; Shao et al. 2018). In some cases, precipitation of the heavy metals are observed upon interaction of the *Spirulina* with the soil which resulted in biosorption of heavy metals (Cepoi et al. 2020).

### Wastewater treatment

Search for potential materials from greener sources for wastewater remediation applications has been crucial for sustainable practices (Zango et al. 2025a, b, 2024a). Various biomass derived from plants, microalgae, natural polymers among others have been explored for wastewater treatments application (Adamu et al. 2024a, b; Garba et al. 2023; Zango et al. 2024a, 2023). Additionally, highly porous synthetic materials such as metal organic frameworks (Afzan et al. 2022; Mahmad et al. 2022; Zango et al. 2021), carbon-based materials (Garba et al. 2015; Hamidon et al. 2024; Zango et al. 2024b), chitosan (Astuti et al. 2025; Zango et al. 2022), metal oxide nanoparticles (Ajil et al. 2023; Zango et al. 2025a, 2025b), etc., have shown great potentials for the wastewater treatment applications. *Spirulina* has emerged important biomass for biosorption of contaminants from wastewater. The remediation process has been attributed to the higher porosity in the *Spirulina* which offers viable alternative for the organic and inorganic uptake from the aqueous medium. It is effective for biosorption of heavy metals, dyes, pharmaceuticals, phenols as well as other inorganic and organic contaminants from multi-component effluents (Vellaiyan 2024; Zhang et al. 2024). Thus, they offer alternative and sustainable solution for tackling the persistent problem due to wastewater contamination (Vellaiyan 2024). The fact that *Spirulina* grows at both hot and alkaline conditions makes it especially useful compared to other species (Diaconu et al. 2023; Mittal et al. 2023). It is investigated for treatment of biosorption of heavy metals, organic contaminants and xenobiotics from wastewaters (Yu et al. 2024). Additionally, it is exploited for the extraction of nutrients from municipal wastewaters and effluents with high amounts of organic matter, which makes it effective against both organic and inorganic compounds. *Spirulina* reportedly reduced dissolved solids, chemical oxygen demand (COD), biochemical oxygen demand (BOD), and total dissolved solids (TDS) (Mittal et al. 2023).

**Fig. 2:**
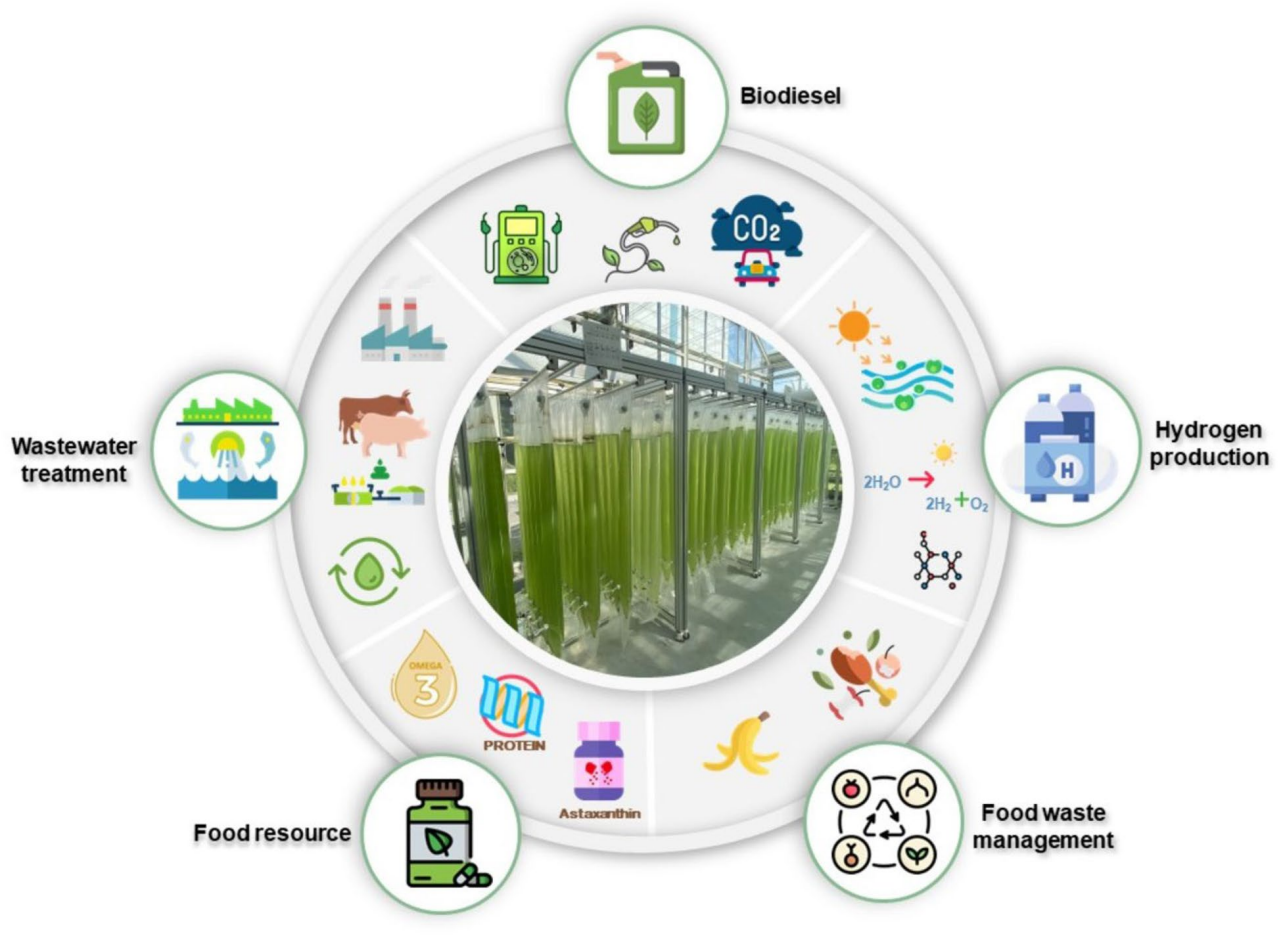
Envisaged circular economy of *Spirulina* for nutraceutical, environmental management and renewable energy applications. Reproduced from Yu et al. (2024); no copyright required

Thus, the *Spirulina* is serving as innovative and promising approach the environmental management, particularly for wastewater remediation. Its potential values as source of renewable energy, higher biosorption efficiency have made it good alternative for circular economy and environmental sustainability applications (Fig. 2**)**. Thus, more research on the benefits of the *Spirulina* is on-going which will significantly help to improve its cultivation condition, harvesting and purification technology as well as processing for optimum consumption, renewable energy and environmental applications to overcome the existing challenges in the field.

#### Biosorption of heavy metals from wastewater

**Fig. 3:**
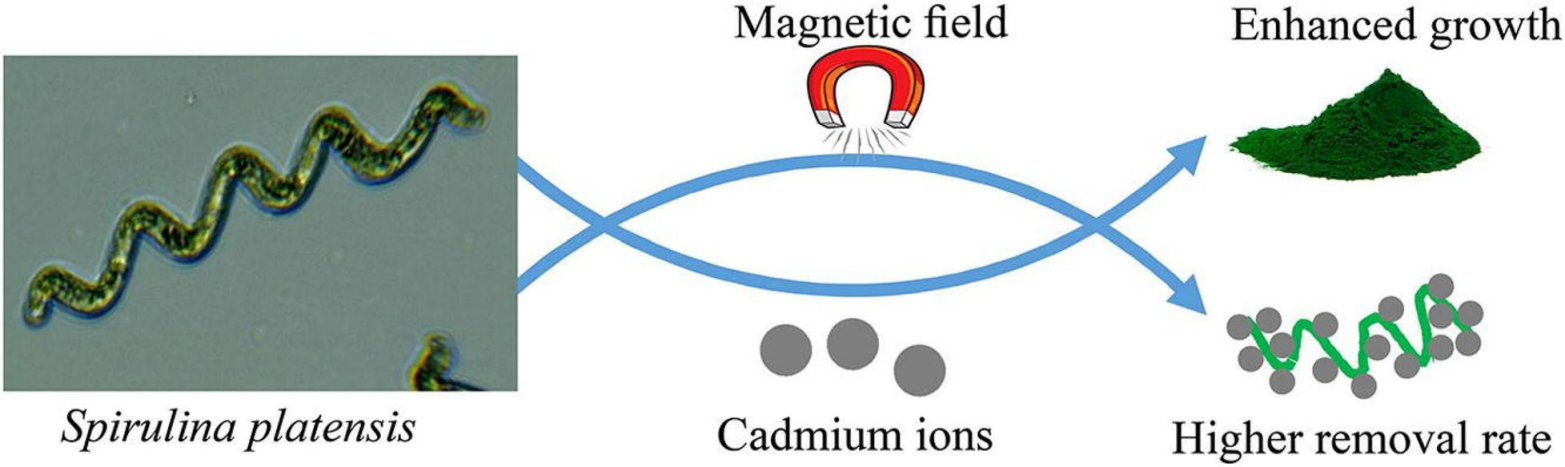
Effect of magnetic field for cultivation of *Spirulina* for heavy metals biosorption. Reproduced from Shao et al. (2018) with permission from Elsevier

The potential exploitations of *Spirulina* for biosorption of heavy metals from wastewaters. Hence, researchers have investigated the efficiency of *Spirulina* for removal of heavy metals. One study discovered the RSM for effective biosorption of Zn (II) by the *Spirulina* (Alharbi et al. 2022). The biosorption efficiency recorded was 97.90% achieved at dose of 4.48 g/L, pH of 6.62 and concentration of 29.72 mg/L for the optimum conditions. The kinetics obeyed pseudo-second order while the isotherm followed Langmuir isotherm model based on monolayer process with adsorption capacity of 50.7 mg/g. Additionally, the capacity of the adsorption was found to increase with temperature which signifies the endothermic and spontaneous nature of the mechanism. Upon reusability, adsorbent has achieved 54%, indicating the efficacy of the algal biomass for the removal application (Alharbi et al. 2022). The effect of static magnetic field (SMF) on the growth and production of *Spirulina* for Cd (II) adsorption was also investigated (Shao et al. 2018). The biomass cultured under 6 h/day SMF significantly enhanced the yield of the *Spirulina*. For the Cd (II) adsorption, an efficiency of 91.4% and 82.3% was achieved at concentration of 10 and 15 mg/L, respectively after 20 days for of biomass culturing under the SMF (Fig. 3). Thus, the work provided innovative technology in which SMF could be employed for the cultivation of the *Spirulina* and its effective for the application for wastewater remediation (Shao et al. 2018).

The use of KCl and Na_2_CO_3_ modified *Spirulina* (SP-KC and SP-NC) and *chlorella vulgaris* (CV-KC and CV-NC) algal biomass was also reported for the heavy metals biosorption (Musah et al. 2022). Under the studied concentration of 25–50 mg/L, removal efficiency of Ni (II) and Fe (II) have reached 100%, whereas removal of Cr (VI) and Cu (II) was in the range of 43.7–74.6% within 120 min, pH of 4 and at room temperature (Musah et al. 2022). Table 3 summarized the findings reported on the application of the *Spirulina* for heavy metals biosorption from wastewater. The optimum conditions for the process have been summarized and the major highlights of the findings have been stated.

**Table 3:** Summary of findings reported on the application of *Spirulina* for biosorption of heavy metals from wastewater

Adsorbent	Pollutant	Optimal condition	Adsorption capacity (mg/g)	Remark	Ref
*Spirulina* sp.	Cd (II) Pb (II)	Concentration = 200 mg/L, dose = 1.5 g/L, pH = 8, T = 30 °C, t = 18 h	184 170	The *Spirulina* sp. has demonstrated higher efficiency for the adsorption of the heavy metals	Bdulkareem and Nwer 2020
*Spirulina platensis*	Pb (II)	Concentration = 20 mg/L, dose = 2 g/L, pH = 10, T = 26 °C, t = 180 min	81.3	The *Spirulina* has achieved higher removal of the Pb (II) with the process best described by Langmuir isotherm model	Izadi and Sadeghi 2023
*Spirulina platensis*	Pb (II)	Concentration = 50 mg/L, dosage = 2 g/L, pH = 7, T = 25 °C, t = 60 min	22.6	The *Spirulina* achieved 92.13 and 84.32% Pb (II) removal in synthetic and real wastewater	Malakootian et al. 2016
*Spirulina platensis*	Cd (II) Pb (II)	Concentration = 1 mg/L, dose = 70 mg/L, pH = 8.8, T = 28 °C, t = 5 days	−	The *Spirulina* has achieved 91.8 and 84.3% removal of the Cd (II) and Pb (II), respectively	Putri et al. 2017
*Spirulina* sp.	Cr (III) Cu (II) Cd (II)	Concentration = 100 mg/L, dose = 1 g/L, pH = 7, T = 35 °C, t = 30 min	185 196 159	The *Spirulina* has demonstrated higher removal of the heavy metals through chemisorption process	Chojnacka et al. 2005
*Spirulina platensis*	Zn (II)	Concentration = 100 mg/L, dose = 0.1 g/L, pH = 8, T = 35 °C, t = 60 min	−	The biosorbent has higher removal efficiency of the Zn (II) at higher concentration up to 300 mg/L	Palaniswamy and Veluchamy 2017
*Spirulina platensis-*polyacrylamide	Pb (II) Cd (II)	Concentration = 30 mg/L, dose = 0.5 g/L, pH = 5–7, T = 25 °C, t = 60 min	337 234	The adsorption efficiency of the *Spirulina* significantly improved by the polyacrylamide	Sun et al. 2019
*Spirulina platensis*	Cd (II) Ni (II)	Concentration = 100 mg/L, dose = 1.0 g/L, pH = 5, t = 350 min	73.6 69.0	The biosorption of the heavy metals occur via monolayer formation	Çelekli and Bozkurt 2011
*Spirulina platensis*	Pb (II) Cd (II) Cu (II)	Concentration = 50 mg/L, dose = 1.0 g/L, pH = 4–6, T = 22 °C, t = 20–25 min	370 201 165	The adsorption efficiency of the *Spirulina* was higher than that of the chlorella for the heavy removal of the heavy metals	Konig-péter et al. 2016
*Spirulina*-alginate bead	Pb (II)	Concentration = 5.63 mg/L, dosage = 50 mg/L, pH = 5.2, T = 25 °C, t = 72 h	114	Immobilization of the *Spirulina* on alginate form biosorbent with higher uptake for the Pb (II)	Villen‐guzman et al. 2021
*Spirulina platensis*-KCl *Spirulina platensis*- Na_2_CO_3_	Ni (II)	Concentration = 100 mg/L, dosage = 0.3 g/L, pH = 4, T = 25 °C, t = 120 min	12.9 13.3	The modified *Spirulina* have been efficient for the Ni (II) and other heavy metals removal	Musah et al. 2022
*Spirulina platensis*-KCl *Spirulina platensis*- Na_2_CO_3_	Fe (II)	Concentration = 100 mg/L, dosage = 0.3 g/L, pH = 4, T = 25 °C, t = 120 min	12.0 11.8	The modified *Spirulina* have been efficient for the Fe (II) and other heavy metals removal	Musah et al. 2022
*Spirulina platensis*	Cr (IV)	Concentration = 50 mg/L, dosage = 0.2 g/L, pH = 1, T = 60 °C, t = 90 min	45.5	The *Spirulina* has removed the adsorbate at both high and low concentrations	Nithya et al. 2019
*Spirulina platensis*	Cu (II) Cd (II) Pb (II)	Concentration = 100 mg/L, dose = 1 g/L, pH = 6, T = 25 °C, t = 90 min	1.00 0.80 0.60	The *Spirulina* beads have achieved complete removal of the heavy metals for five reusable cycles	Kőnig-Péter et al. 2016
*Spirulina platensis*	Cd (II)	Concentration = 60 mg/L, dose = 2 g/L, pH = 8, T = 26 °C, t = 90 min	−	The biosorbent has achieved 97% removal efficiency within 90% of the adsorption process	Al-Homaidan et al. 2015
*Spirulina platensis*	Al (III) Ni (II) Cu (II)	Concentration = 50 mg/L, dose = 2.5 g/L, pH = 6, T = 25 °C, t = 120 min	41.0 42.6 38.9	The *Spirulina* has achieved higher adsorption of the heavy metals as governed by pseudo-second order and Langmuir model	Almomani and Bohsale 2021
chitosan/*Spirulina* films	Pb (II)	Concentration = 50 mg/L, pH = 6, T = 25 °C, t = 24 h	63.3	The chitosan presence has significantly improved the adsorption performance of the *Spirulina*	Yahia et al. 2024
*Spirulina* sp.	Co (II)	Concentration = 50 mg/L, dose = 1 g/L, pH = 6, T = 45 °C, t = 1200 min	95.9	The *Spirulina* mas has achieved higher efficiency then AC for the Co (II) adsorption	Peres et al. 2018
*Spirulina platensis*	Cr (IV)	Concentration = 50 mg/L, dosage = 1 g/L, pH = 3, T = 25 °C, t = 30 min	578	Higher adsorption of the 99.8% was achieved, attributed to higher S_BET_ of the *Spirulina*	Gunasundari and Kumar 2017
*Spirulina platensis*	Cr (IV) Fe (II) Cu (II)	Concentration = 50 mg/L, dosage = 100 mg/L, pH = 9.5, T = 25 °C, t = 30 min	4.53 3.93 3.01	The *Spirulina* has demonstrated higher uptake of the heavy metals from the effluents at different pH of the solution	Zinicovscaia et al. 2019
Ultrasonic *Spirulina platensis* S-modified *Spirulina platensis*	Cu (II)	Concentration = 500 mg/L, dosage = 1 g/L, T = 25 °C, t = 90 min	775.3 446.8	The ultrasonic and sulfur modification of the *Spirulina* has significantly enhanced its adsorption efficiency for the adsorption of heavy metals	Gunasundari 2017

#### Biosorption of dyes from wastewater

The higher surface area of the *Spirulina* and its filamentous structure have manifested in its ability to interact with dye molecules via physical and chemical process. The biosorption process is favored by electrostatic or ionic, van der Waals forces and hydrogen bonding interactions (Ayachi et al. 2019). The functional moieties like carboxyl, amine, and hydroxyl on the cell surface of the *Spirulina* enhances its binding affinity to wide spectrum of cationic, anionic, and neutral dyes. The cationic dyes such as malachite green, methyl orange, methylene blue, crystal violet etc., (Buhani et al. 2019). effectively interact with the sites containing negatively charged molecules on the *Spirulina* surface, whereas the anionic dyes such as reactive dyes, congo red, eosin adsorbed onto the surface of the *Spirulina* via electrostatic interactions or hydrogen bonding interactions (Mohadi et al. 2017).The effectiveness of the *Spirulina* for the dyes biosorption is dependent on factors such as the dyes concentration, contact duration, adsorbent dose, temperature, pH, ionic strength and other parameters to be optimized.

Thus, researchers have investigated the biosorption of wide spectrum of dyes on both *Spirulina* strains. Al Hamadi et al., reported on the biosorption of azo dyes onto the *Spirulina* (Hamadi et al. 2017). Using the adsorbate concentration of 100 mg/L, pH of 2, biosorbent dosage of 0.5 g/L and contact time between 60–75 min, the removal efficiency achieved was 98.55 and 97.05% for the Acid Black 210 and Acid Blue 7, respectively. Meanwhile, the process has been favored by the increase in temperature from 35–60 °C which replicated the adsorbate molecules mobility and the abundant pores existence on the *Spirulina* surface for uptake of dye molecules (Hamadi et al. 2017). The biosorption property of the *Spirulina* was also investigated for tartrazine and FD&C red no. 40 anionic dyes (Ben Torkia et al. 2018). It was observed that increased in the acidity of the solution improved the dyes adsorption. Thus, at acidic pH, the negatively charged anions of dyes and the hydroxyl (OH^−^) are attracted to the adsorbate receptor sites. When the pH value is decreased, the solution H_3_O^+^ increased and the OH^−^ become replaced by the anionic dyes. Similarly, at higher pH of the solution, the concentration of quantity of OH^−^ increase which compete with the anionic dyes for the receptor sites that causes the decreased in the adsorption efficiency. On the other hand, increasing the temperature decreased the interaction between the dyes and the receptor sites due to increase in thermal collision. Thus, the adsorption capacity achieved for the tartrazine and FD&C red no. 40 anionic dyes was 23.66 and 228.05 mg/g, respectively, at pH of 4 and temperature of 25 °C (Ben Torkia et al. 2018).

Modified forms of the *Spirulina* have been considered due to their enhanced surface features and functionalities which offers higher adsorption performance and stability for the composite biosorbents. For instance, layered double materials have been functionalized onto *Spirulina* for the formation of Ni–Al-*Spirulina* (NiAl-Sp) and Zn-Al-*Spirulina* (ZnAl-Sp) for selective adsorption of cationic dyes (Fig. 2) (Lesbani et al. 2024). Thus, for the malachite green adsorption, a Langmuir adsorption capacity of 478.19 and 123.457 mg/g has been achieved by the NiAl-Sp and ZnAl-Sp, respectively. The mechanism for the adsorption is governed not only by electrostatic interactions but also hydrogen bonding which existed between the malachite green and the amino, hydroxyl, and carboxyl functional moieties originating from the *Spirulina*. During the adsorption, substantial number of π-π interactions occurred between the aromatic rings of malachite green and the adsorbents (Lesbani et al. 2024). Table 4 highlights various findings on the usage of *Spirulina* biomass for the adsorption of dyes from wastewaters. The conditions for the optimum adsorption capacity and the major findings were summarized.

**Table 4:** Summary of findings reported on the application of *Spirulina* for biosorption of dyes from wastewater

Adsorbent	Pollutant	Optimal condition	Adsorption capacity (mg/g)	Remark	Ref
*Spirulina platensis*	Tartrazine FD&C red no. 40	Concentration = 600 mg/L, dosage = 50 mg/L, pH = 4, T = 25 °C, t = 24 h	23.7 228	The adsorption performance is attributed to the interaction between the anionic dyes and receptor sites on the *Spirulina* surface	Ben Torkia et al. 2018
*Spirulina platensis*	Indigo blue	Concentration = 100 mg/L, dose = 1 g/L, pH = 4, T = 50 °C, t = 96 h	89.9	The *Spirulina* has demonstrated higher adsorption of the dye which was favored by higher temperature and acidic pH of the solution	Robledo-Padilla et al. 2020
*Spirulina platensis*	Reactive red 120	Concentration = 50 mg/L, dose = 50 mg/L, pH = 2–3, T = 25 °C, t = 6 h	482	The *Spirulina* has demonstrated superior adsorption than the activated carbon employed for comparison	Cardoso et al. 2012
*Spirulina platensis*	Ismate violet 2R	Concentration = 10 mg/L, dosage = 0.1 g/L, pH = 6, T = 25 °C, t = 120 min	14.7	The *Spirulina* has shown higher adsorption efficiency for the removal of the dye in both simulated and actual wastewater samples	Alprol et al. 2021
*Spirulina platensis*	Direct yellow 12	Concentration = 100 mg/L, dosage = 0.1 g/L, T = 30 °C, t = 2000 min	714	The *Spirulina* has achieved higher adsorption capacity for the dye adsorption as favored by Temkin model	Marzbali et al. 2017
*Spirulina platensis*	Methylene blue	Concentration = 100 mg/L, dosage = 0.5 mg/L, pH = 7, T = 25 °C, t = 240 min	312	Higher adsorption capacity has been achieved at natural and atmospheric temperature of the solution	Mitrogiannis et al. 2015
*Spirulina platensis*	Basic red 46	Concentration = 50 mg/L, dose = 0.05 mg/L, pH = 6, T = 30 °C, t = 70 min	24.5	The adsorption was favored by Langmuir model with adsorption capacity of 24.46 mg/g	Deniz and Kepekci 2016
*Spirulina platensis*	Reactive red 120	Concentration = 50 mg/L, dose = 50 mg/L, pH = 2, T = 25 °C, t = 6 h	482	The adsorption efficiency of the *Spirulina* was higher than the AC compared, removing 97.1% of the dye	Gally et al. 2012
*Spirulina platensis*	Malachite green	Concentration = 100.54 mg/L, dosage = 0.98 g/L, pH = 7.57, t = 52.53 min	103	Maximum adsorption efficiency of 94.1% was achieved according to Box-Behnken design	Bonyadi et al. 2022
*Spirulina platensis*	FD&C red no. 40 Acid blue 9	Concentration = 500 mg/L, dosage = 250 mg/L, T = 25 °C, t = 120 min	295 1.45 × 10^3^	The biosorption of the dyes onto the *Spirulina* was attributed to higher porosity of the nanoparticles	Dotto et al. 2012a
*Spirulina platensis*	FD&C red no. 40 Acid blue 9	Concentration = 500 mg/L, dosage = 250 mg/L, T = 25 °C, t = 120 min	400 1.65 × 10^3^	Box-Behnken design was employed for the optimization of the adsorption process	Dotto et al. 2012b
*Spirulina platensis*	Tartrazine Allura red	Concentration = 10 g/L, dosage = 50 mg/L, pH = 4, T = 25 °C, t = 120 min	363 469	The biosorption of the dyes was favored by lower temperature and acidic pH of the solution	Dotto et al. 2013
*Spirulina* sp.	Procion red Congo red	Concentration = 5 mg/L, dosage = 0.5 g/L, pH = < 7, T = 25 °C, t = 70 min	11.2 0.148	The dyes adsorption was faster, and the process proceeds via chemical adsorption	Mohadi et al. 2017
*Spirulina* sp.	Crystal violet Methylene blue	Concentration = 100 mg/L, dosage = 50 mg/L, pH = 8, T = 27 °C, t = 60 min		Higher adsorption of the dyes was achieved up to five cycles which was favored by Freundlich model	Buhani et al. 2019
NiAl- *Spirulina platensis* ZnAl- *Spirulina platensis*	Malachite green	Concentration = 80 mg/L, dosage = 200 mg/L, pH = 4, T = 30 °C, t = 30 min	478 123	The *Spirulina* composites have demonstrated higher S_SBET_ for adsorption of the dye	Lesbani et al. 2024
*Spirulina* *Spirulina*/chitosan	Methylene blue	Concentration = 500 mg/L, dosage = 500 mg/L, pH = 8, T = 25–55 °C, t = 24 h	445 336	Both adsorbents have shown higher efficiency for the methylene blue adsorption	Sellaoui et al. 2024
*Spirulina maxima-*ZnCl_2_ *Spirulina maxima-*H_3_PO_4_	Methylene blue	Concentration = 300 mg/L, dose = 2 g/L, pH = 6, T = 31 °C, t = 4 h	344 292	The modified *Spirulina* has shown superior performance for the dye adsorption	Lebron et al. 2019
HDTMA-modified *Spirulina* sp.	Crystal violet Safranin	Concentration = 50 mg/L, dose = 5 g/L, pH = 2, T = 25 °C, t = 3 h	102 54.0	The modification has significantly improved the adsorption performance of the *Spirulina* biomass	Guler et al. 2016
*Spirulina* sp.-silica *Spirulina* sp.-silica/magnetite	Methylene blue	Concentration = 100 mg/L, dose = 50 mg/L, pH = 6, T = 25 °C, t = 60 min	80.3 90.9	The functionalization significantly enhanced the *Spirulina* performance for the adsorption of methylene blue	Kausar and Buhani 2020
*Spirulina maxima* *Chlorella pyrenoidosa*	Methylene blue	Concentration = 100 mg/L, dose = 1 g/L, pH = 6, T = 28 °C, t = 150 min	145 114	The *Spirulina* has demonstrated higher adsorption according to the Langmuir monolayer isotherm	Lebron et al. 2018
*Spirulina*/chitosan	Malachite green	Concentration = 4 mg/L, dose = 112.5 mg/L, pH = 6.8, t = 93.75 min	3.23	Over 99% of the dye was removed by the composite at optimum adsorption condition	Mousavi et al. 2023
*Spirulina* immobilized alginate	Tartrazine Carmoisine Allura red	Concentration = 50 mg/L, dosage = 200 mg/L, pH = 3, t = 1 h	76.9 50.0 71.4	The immobilization significantly enhanced the *Spirulina* adsorption efficiency for the removal of dyes	Saloglu and Irmak 2021

## Biodiesel production

The fact that *Spirulina* contains lipids (around 6.4–14.3% of its dry weight) has made it a vital source of industrial raw materials and feedstock. Prior, biodiesel production was solely dependent on first generation feedstocks such as peanut, palm, rapeseed, soybean, etc., that at the same time used as vital sources of food. As a result, there evolved competition in food supply and energy demands from the plants sources (Lopes et al. 2023). Thus, the needs for the second-generation feedstock for the biodiesel from non-edible sources (Shirazi et al. 2017). The *Spirulina* served as an alternative source to produce the biodiesel (Murad and Al-Dawody 2020; Shirazi et al. 2017). Its efficient harvesting methods, fast growing rate and cultivation flexibility has made it a vital source for the biodiesel production. It could be extracted and transformed into biodiesel via transesterification process. The biodiesel products of the micro algal biomass are non-toxic, renewable, and biodegradable with potential to replace petroleum-based fuel in CI engines (Kumar et al. 2022a, b). The algal biomass grows rapidly under variety of conditions including suboptimal nutrient environments. The *Spirulina* protein and lipid content makes it as well as high cultivation yield makes it attractive and alternative microalga for large-scale production (Vellaiyan 2024). Its cultivation has been more economical and environmentally sustainable than other microalga that are used for the biodiesel production (Fattah et al. 2020; Neag et al. 2022). Moreover, it has less water and farm size requirements in addition to the carbon dioxide consumption and emission (Murad and Al-Dawody 2020).

The process of utilization of microalga to produce biodiesel has recently garnered more traction among the researchers (Rawindran et al. 2023; Zango et al. 2019; Zulfadhli et al. 2024). In comparison to petroleum fuels which are known for the large emission of NO_x_ and SO_X_ and, the microalga are favourable for combustion with less emission of atmospheric pollutants and low energy out-put (Muhamad et al. 2023; Murad and Al-Dawody 2020). Most importantly, the power they generated is equal generates equal in intensity to that of the petroleum products (Murad and Al-Dawody 2020). The lipids extraction process in the biodiesel is usually complex and consumed high energy. Thus, efficient technologies have emerged which are cost effective.

**Fig. 4:**
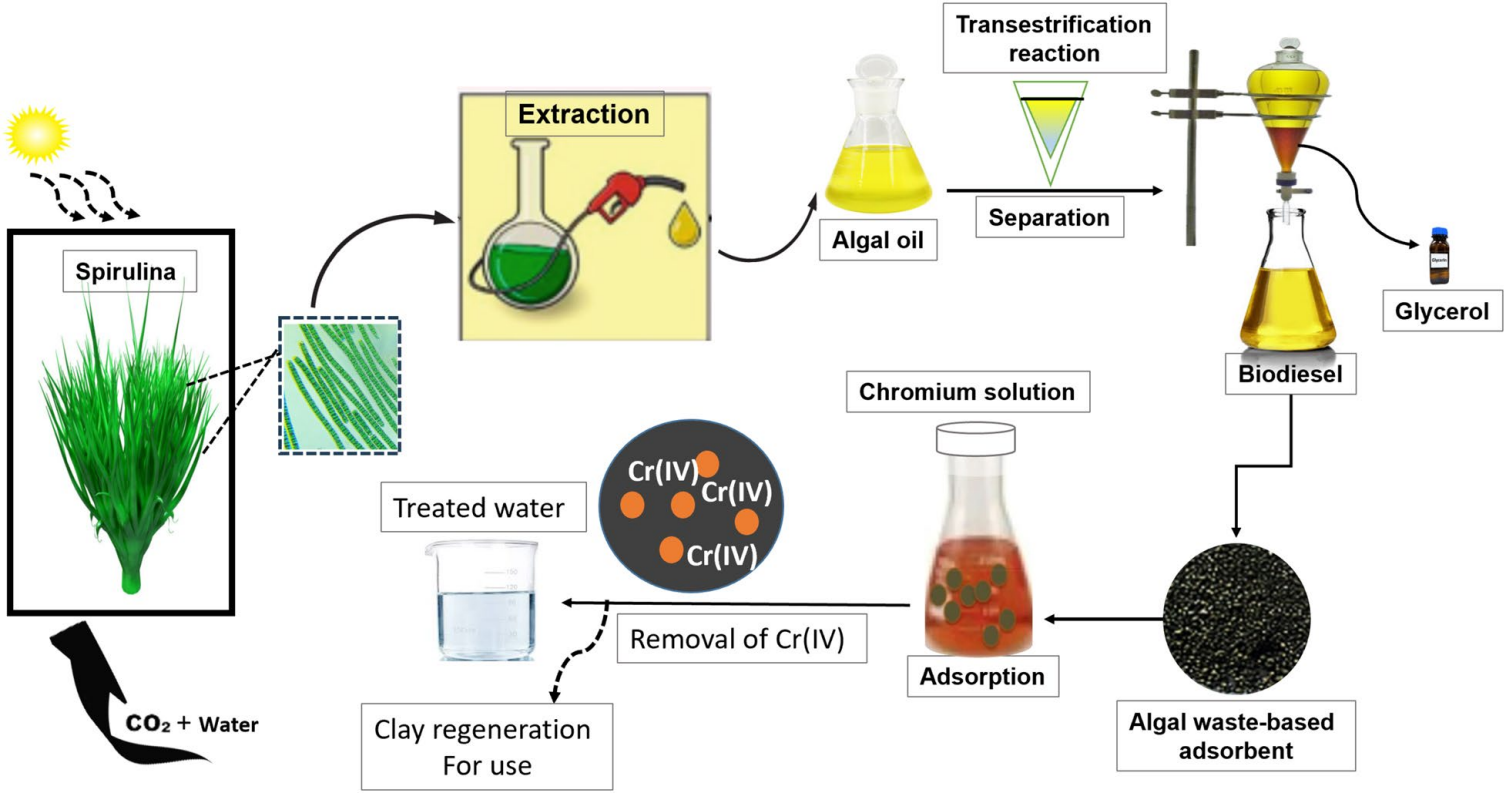
*Spirulina* cultivation and its application as feedstock for biodiesel production and adsorbent for wastewater remediation

Researchers leverages the use of *Spirulina* due to its lipid content. It served as promising feedstock that employed esterification and transesterification conversion techniques for the biodiesel production (Al-Dawody et al. 2022; Haghighi et al. 2022). The processes proceed via two steps. In the first step, the microalga are extracted using both mechanical and chemical methods (Zhou et al. 2022). Various solvents such as methanol, ethanol, acetone, and sulfuric acid are used. Sometimes, solvents combination is more effective for the extraction process compared to the single solvent (Kusmiyati et al. 2020; Rahman et al. 2017). The second step involved the esterification and transesterification process. The conversion process is taking place with the help of an acidic or basic catalyst, with the production of the biodiesel and glycerol as major products (Murad and Al-Dawody 2020; Mohamadzadeh Shirazi et al. 2017). The residue of the biomass could be use as biosorbent for the contaminants adsorption from the wastewater as demonstrated in the Fig. 4.

Thus, various researchers have reported on the exploitation of *Spirulina* for biodiesel production. Shirazi et al., scrutinized on the exploitation of *Spirulina* as feedstock for the biodiesel production via transesterification process. A response surface methodology was employed according to the central composite design for the lipid extraction and subsequent conversion to the biodiesel under supercritical methanol condition (Shirazi et al. 2017). The independent factors adopted as the most important parameters for the extraction include the temperature (°C), extraction time (min), methanol-to-dry *Spirulina* (mL/g), hexane-to-dry *Spirulina* (mL/g), and moisture content (%). Accordingly, a conversion efficiency of 99.32% for the fatty acid methyl esters (FAME) was obtained underneath the optimum condition, the temperature of 300 °C, extraction time of 30 min, methanol-to-dry *Spirulina* of 8 mL/g, hexane-to-dry *Spirulina* of 4 mL/g, as well as moisture content of 40% (Shirazi et al. 2017).

*Spirulina*'s carbohydrate content can also be converted into bioethanol through fermentation processes. Anaerobic digestion of *Spirulina* biomass can produce biogas, primarily composed of methane (Astolfi et al. 2020; Rempel et al. 2019a). This process not only generates renewable energy but also helps in managing waste biomass. Studies have indicated that *Spirulina* can be effectively utilized in biogas production, contributing to sustainable energy generation (Lakatos et al. 2019; Werlang et al. 2020). While the *Spirulina* lipid content is low compared to other microalgae such as Chlorella, certain cultivation strategies can enhance its lipid accumulation, making it a potential candidate for the biodiesel production (Saeedi Dehaghani and Pirouzfar 2018). Research has demonstrated that under specific stress conditions, *Spirulina* lipid content can be increased which can be trans-esterified into biodiesel (Can et al. 2017a; Shirazi et al. 2017). Hydrogen gas is considered a clean energy carrier, and certain strains of *Spirulina* have shown the ability to produce hydrogen under specific conditions. The presence of hydrogenases the *Spirulina* enables hydrogen generation which presents another avenue for bioenergy applications (Ainas et al. 2017; Saka et al. 2020).

Pradana et al., reported that primary constituents of the biodiesel produced by the *Spirulina* include methyl oleate and methyl palmitate with yields of 45.10% and 41.03% respectively. While the yield for methyl linoleate and methyl stearate are 8.34% and 5.54%, respectively. Hexane coextraction and increased temperature can enhance its biodiesel yield while increased methanol levels does the opposite (Pradana et al. 2018). The residual biomass after lipid extraction can be repurposed as high-protein animal feed, enhancing the overall economic viability of *Spirulina* cultivation for biodiesel. Table 5 highlights various findings on the usage of *Spirulina* biomass for the biodiesel production. Ongoing research is focused on improving the strains of *Spirulina* for higher lipid yields, optimizing cultivation conditions. Even though the *Spirulina* has presented promising and vital avenue to produce biodiesel, more research are needed to explore technological advancements for its cultivation. Additionally, feasibility studies are needed in terms of economic analyses that will help in boosting its commercialization and integration into the biofuels market.

**Table 5:** Summary of findings on the application of *Spirulina* for biodiesel production

Feedstock	Extraction process	Optimal condition	Total FAME yield (%)	Remark	Reference
*Spirulina platensis*	Solvent extraction	Temp = 300 °C, extraction time = 30 min, methanol-to-dry *Spirulina* = 8 mL/g, hexane-to-dry *Spirulina* = 4 mL/g, moisture content = 40%	99.3	Higher yield of biodiesel (over 99%) was obtained according to the RSM analysis	Mohamadzadeh Shirazi et al. 2017
*Spirulina platensis*	Methanol extraction	Microalga vol = 100 mL, methanol vol = 25 mL, catalyst dosage = 2 g, temp = 65 °C,	78.4	The major FAME obtained include linoleic, Oleic, and stearic acid methyl esters (AME)	(Murad and Al-Dawody 2020)
*Spirulina* sp.	Methanol extraction	Catalyst dosage = 0.15 g, *Spirulina* oil-to-methanol volume ratio = 1:3, temp. 80 °C, extraction time = 48 h	98.4	The major product obtained include linoleic, palmitoleic, palmitic, margaric, oleic, arachidic, behenic, lignoceric, and tricosanoic AME	Haghighi et al. 2022
*Spirulina maxima*	Methanol/sulfuric acid extraction	Catalyst concentration = 0.75 wt.% KOH, methanol to oil ratio = 12:1, temp. 65 °C, extraction time = 90 min	86.1	The major fatty acids obtained include palmitic (40.2%), linoleic (17.9%), linolenic (18.3%) AME	Rahman et al. 2017
*Spirulina* sp.	Ethanol extraction	Catalyst = 60%CaO/Al_2_O_3_, ethanol to lipid molar ratios = 24:48, temp. 50 °C, extraction time = 30 min, and pressure = 1.0 atm	99.0	Under the optimum condition, higher quality ethyl ester biodiesel (yield 90–99%) was obtained	Turkkul et al. 2020
*Spirulina platensis*	Single stage extraction	*Spirulina* drying = 90 min, catalyst concentration = 60%, 1:4 *Spirulina* to methanol ratio = 1:4, stirring intensity = 450 rpm, temp. = 55 °C	75.0	The fatty acid obtained are palmitic (41.2%), linolenic (17.8%), linoleic (12.6%), oleic (4.11%), caprylic (3.90%) and palmitoleic (3.39%) AME	Nautiyal et al. 2014
*Spirulina* sp.	Solvent extraction	Mass of extract biomass = 30 mg, catalyst concentration = 0.05 M (MeONa), temp = 100 °C, extraction time = 10 min	87.6	The higher lipid content (32.7%) of the *Spirulina* has resulted in the high yield of the biodiesel produced	de Morai et al. 2018
*Spirulina* sp.	n-hexane extraction	Catalyst dosage = 700 mg, methanol to oil ratio = 7:1, temp. 60 °C, extraction time = 4 h	95.0	A 95% yield of the FAME was obtained at the optimal condition with high efficiency for four reusable cycles	Taherinia et al. 2024
*Spirulina* sp.	Methanol extraction	Catalyst dosage = 0.2 g, mass of *Spirulina* = 150 g, methanol to n-hexane ratio = 7:1, temp. 50 °C, extraction time = 4 h	100	The fatty acids obtained include methyl palmitat (41.0), methyl linoleate (8.34), methyl oleate (45.0) and methyl stearate (5.54)	(Surya Pradana et al. 2018)
*Spirulina maxima*	Hexane/iso-propanol extraction	Catalyst dosage = 6 g, methanol to oil ratio = 6:1, temp. 65 °C, extraction time = 60 min	96.6	High yield of FAME was obtained which include mistic, palmitic, palmitoleic, steric, oleic, linoleic acids	Murthy and Kumar 2021
*Spirulina platensis*	Solvent extraction	Mass of extract biomass = 170 g/L, catalyst concentration = 0.2 mol/L (sodium phosphate), temp = 100 °C, extraction time = 24 h	82.0	The conversion process has produced large quantity of biomethane with higher energy potential	Rempel et al. 2019b
*Chlorella* sp.	Microwave-assisted extraction	Mass of extract biomass = 10 g/L, dry *Spirulina* to methanol ratio = 1:12, catalyst concentration = 1%, microwave irradiation power = 450 W, extraction time = 50 min	93.4	The microwave-assisted technique has significantly improved the production of the biodiesel from the *Chlorella* sp., resulting in the high yield of the FAME	Ummu Kalsum et al. 2019b, a
*Spirulina platensis*	Microwave-assisted extraction	Mass of extract biomass = 10 g/L, dry *Spirulina* to methanol ratio = 1:12, catalyst concentration = 1%, microwave irradiation power = 800 W, extraction time = 40 min	83.7	The microwave-assisted technique has significantly improved the production of the biodiesel from the *Spirulina* sp., resulting in the high yield of the FAME	Ummu Kalsum et al. 2019b, a
*Spirulina platensis*	Microwave-assisted extraction	Oil to methanol ratio = 1:6, temp = 65 °C, time = 50 min	88.0	High yield of the biodiesel was obtained by the microwave-assisted technique with 88% yield	Purnama et al. 2020
*Spirulina platensis*	–	Catalyst dosage = 0.2 g, mass of *Spirulina* = 0.03 g, methanol to n-hexane ratio = 7:1, temp. 60 °C, extraction time = 4 h	100.0	Higher yield of the FAME was obtained with the major ones such as linoleic (23.4%), trans linolenic (20.8%) and palmitic (32.7%) acids	Seyhaneyildiz Can et al. 2017b
*Spirulina platensis*	Methanol/chloroform extraction	Biomass ratio = 12:1 v/w, methanol to chloroform ratio = 2: 1, catalyst concentration = 11.9%, extraction time = 22 min	92.3	The FAME yield was increased by the increase in ultrasound irradiation	Kusmiyati et al. 2020
*Spirulina* sp.	Methanol extraction	Catalyst loading = 3wt.%, methanol to oil ratio = 30:1, temp. 80 °C, extraction time = 3 h	99.0	Under the optimal condition, 99% efficiency has been achieved with the nanocatalyst sustained activity up to 5 cycles	Mittal and Ghosh 2023
*Spirulina* sp.	chloroform/methanol extraction	Mass of extract biomass = 7 g/L, catalyst dosage = 0.05 g temp. 45 °C, extraction time = 3 h	79.5	The major FAME identified include methyl palmitate (39.2%) and methyl cis-9-Octadecenoate (39.0%)	Sumprasit et al. 2017a

Despite its potential, several challenges need to be addressed for large-scale bioenergy production using *Spirulina*. Such challenges include enhancing lipid accumulation and carbohydrate content which requires precise control of growth conditions. Developing cost-effective harvesting and processing techniques is crucial for commercial applications with advancements in genetic tools having the ability to further improve *Spirulina*'s bioenergy yields and stress tolerance.

## *Spirulina*-based biotemplate

The biotemplating process has been described as the use of biological entities such as protein, enzyme, or microorganism to fabricate a template or scaffold that can be used for the synthesis or assembly of organic and inorganic materials. The structure and functional properties of biomaterials are often leveraged to guide the desired materials formation with specific features. Thus, the process of biotemplating has many advantages for materials synthesis with biocompatibility and versatility for internal applications (Gong et al. 2018; Hosseini et al. 2020). They present good control and precision over the composition, properties, size, shape of the materials synthesized. The materials synthesized using biotemplates are suitable for the application in biomedical science because of their biocompatibility. The friendly nature of the biotemplate materials in comparison to other materials synthesized using conventional methods is also of advantages, particularly in a situation where the principle of green chemistry is required. Thus, the biotemplating processes offers sustainable material production practices than the conventional techniques (de la Asunción-Nadal et al. 2022).

**Fig. 5:**
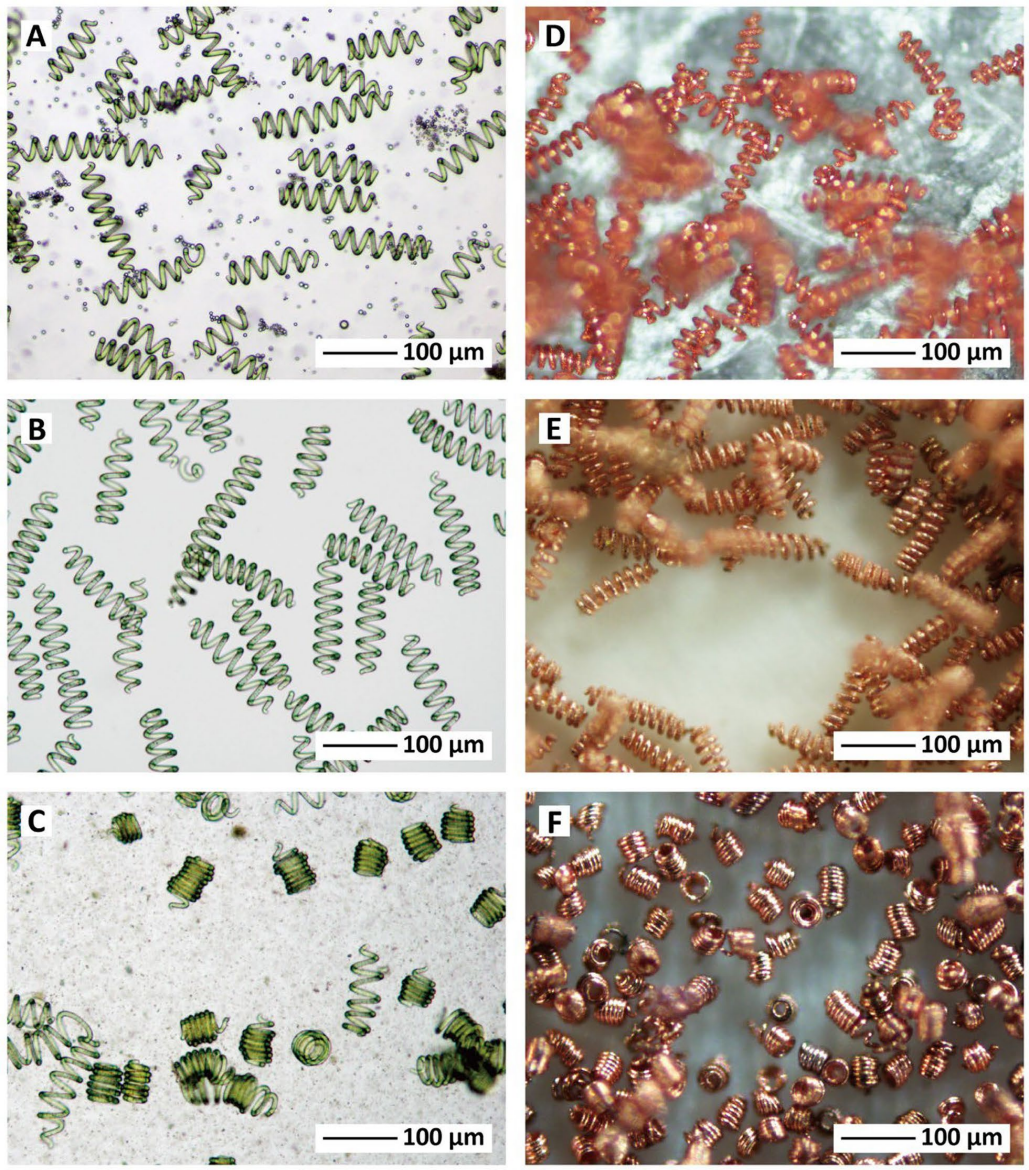
**a**–**c** Right-handed (RH) *Spirulina* and their biotemplate products **d** RH µcoils, 19 mm; **e** RH µcoils, 14 mm; **f** RH µcoils, 6 mm. Adopted from Kamata et al. (2014); no copyright required

The use of *Spirulina* for biotemplating process has been investigated. The vital features of the microalga such as its higher porosity and friendly nature are utilized to for the formation of desired products. Researchers have studied its application as biotemplate for wide range of products such as in the nanomaterials, biosensors, and biocompatible scaffolds for tissue engineering applications (Meng et al. 2018)(Zheng et al. 2021). Kamata et al. employed the *Spirulina* for the design of biotemplate by electroless plating method technique (Kamata et al. 2014). The process results in smooth metal layer formed on the surface of the targeting object to be plated stages such as the fixation of *Spirulina*, Pd catalysation (Pd nanoparticle adsorption as plating catalyst nuclei), and copper electroless plating are involved. The left hand (LH) and right hand (RH) biotemplate have been conducted in plating bath using a cultivation medium with 10^5^ m/L *Spirulina* concentration, producing roughly two million µcoils (90 mg, 80% yield) The µcoils has been characterized by XRD, XPS, SEM and ICP. The optical micrographs of the fabricated LH and RH µcoils are shown in Fig. 5. In L_free_/N, the RH *Spirulina* was more tightly coiled and smaller than around 20 mm. The identical process used for the LH series was used to prepare the RH templates and matching µcoils.

**Fig. 6:**
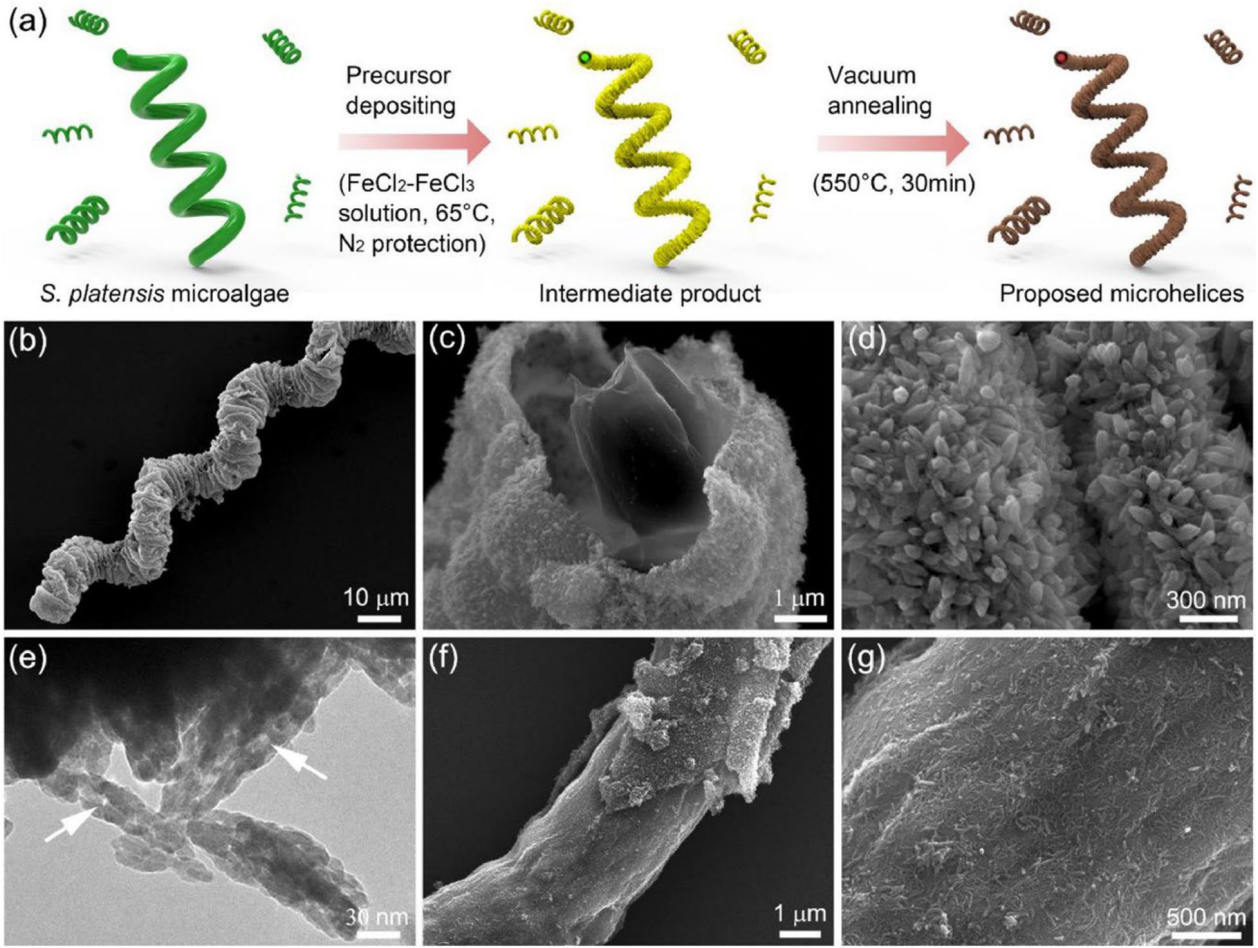
*Spirulina* biotemplate **a** fabrication process. **b**–**d** FESEM images, showing the surface morphology. **e** High-magnification TEM image of individual spindle NP presented in (**d**). The white arrows indicate mesoscale pores. **f**, **g** Low- and high-magnification FESEM images of the core depicted in (**c**). Reproduced from Zheng et al. (2021) with permission from Elsevier

By utilizing the *Spirulina*'s natural structure and properties, scientists can design and control the fabrication of materials with specific properties and functionalities. Thus, Zheng et al., demonstrated the fabrication of core–shell-structured hollow helical microswimmer via a straightforward *Spirulina*-based biotemplating process of low cost and high yield (Zheng et al. 2021). The process for the fabrication as well as the FESEM and TEM of the biotemplate was shown in Fig. 6. The microswimmer obtained has porous carbon structure with the inner and outer core shell aggregated on the surface of magnetite nanoparticles (NPs) with mesoporous spindle-like structure. Moreover, the desirable features of high surface area and photothermal attributes have been integrated which form vital part of the material for medicinal applications. It has been applied for biological detoxification, photothermal antibacterial therapy, and drug delivery for gastrointestinal tract applications. Also, its biosorption property of the *Spirulina*-based biotemplate for heavy metals remediation has been reported (Zheng et al. 2021). Overall, *Spirulina* offers sustainable environment for fabricating biocompatible structures for various applications in fields such as biotechnology, nanoscience, and environmental remediation (Gong et al. 2018; Karami-Osboo et al. 2022).

### Economic feasibility, techno-economic and life cycle assessment of *Spirulina*

A comprehensive techno-economic assessment of *Spirulina* across diverse applications reveals its significant potential and challenges, highlighting the importance of optimizing production processes and exploring innovative applications to enhance its economic viability and sustainability. In the nutraceutical sector, *Spirulina's* rich composition of proteins, vitamins, minerals, and antioxidants positions it as a valuable dietary supplement, yet the cost of production, extraction, and formulation can influence its market competitiveness; therefore, techno-economic assessments in this area often focus on optimizing cultivation methods, improving biomass yield, and developing cost-effective extraction techniques to ensure the affordability and accessibility of *Spirulina*-based nutraceutical products (Marjanović et al. 2024). The medicinal applications of *Spirulina*, driven by its bioactive compounds with anti-inflammatory, antioxidant, and immunomodulatory properties, also necessitate rigorous techno-economic evaluation to justify the investment in research, clinical trials, and the development of standardized extracts; such assessments would typically consider the cost of isolating and purifying specific bioactive compounds, the efficacy of these compounds in clinical settings, and the potential market size for *Spirulina*-derived pharmaceuticals (Spínola et al. 2024b). From an environmental perspective, *Spirulina* offers promising solutions for wastewater treatment and carbon sequestration, but the economic feasibility of these applications depends on factors such as the scale of operation, the efficiency of nutrient removal, and the potential for biomass valorization; techno-economic studies in this context may evaluate the cost-effectiveness of using *Spirulina* cultivation systems for wastewater treatment compared to conventional methods, the potential revenue from selling the harvested biomass, and the environmental benefits in terms of reduced pollution and greenhouse gas emissions (Rodríguez et al. 2018). In the realm of bioenergy, *Spirulina's* potential as a feedstock for biofuel production has been explored, with techno-economic analyses playing a crucial role in determining the economic viability of converting *Spirulina* biomass into biofuels like biodiesel and bioethanol; these assessments usually involve evaluating the costs associated with biomass production, lipid extraction, and the conversion process, as well as comparing the energy output and economic returns with those of traditional fossil fuels.

*Spirulina's* high protein (60–70%), essential nutrients, and antioxidants make it ideal for nutraceuticals, requiring thorough techno-economic analysis for viable production. Open raceway ponds offer low-cost cultivation (5–12/kg) but face contamination risk, while photoreactor ensure premium based on quality biomass (10–25/kg) at higher capital costs of at least 700,000–2 M/ha. Optimal growth requires controlled light, temperature, pH, and nutrients, with phycocyanin content significantly influencing end-product value (Gao et al. 2020). Harvesting via filtration or centrifugation impacts operational costs, followed by energy-intensive drying (spray drying at 15–30/kg vs. freezing dying at 50–100/kg) (Demirbas and Edris 2017). Optimal growth requires controlled light, temperature, pH, and nutrients, with phycocyanin content significantly influencing end-product value (Gao et al. 2020). Harvesting via filtration or centrifugation impacts operational costs, followed by energy-intensive drying (spray drying at 15–30/kg vs freezing-drying at 50–100/kg) (Seghiri et al. 2021). Downstream extraction of high-value compounds like phycocyanin (100 − 500/g) boosts profitability and drives demand for Spirulina powders (15 − 50/kg), capsules(15 − 50/*kg*), *capsules* (20–100/bottle), and extracts, with production scalability (5–50 ha farms yielding 1 M − 1* M* − 10 M annually) and return on investment (ROI) in 3–7 years (Chaiklahan et al. 2018). However, sustainability is enhanced by low water use, CO₂ sequestration, and non-arable land cultivation, though energy-efficient drying and green extraction need optimization. Also, regulatory compliance (e.g., FDA, EU standards) and clinical validation of health claims are crucial for market access. Upon all these, strategic investments in PBRs, diversified products, and direct-to-consumer models maximize margins, positioning *Spirulina* as a sustainable, high-value nutraceutical resource.

The medicinal potential of *Spirulina*, driven by bioactive compounds like *phycocyanin*, polysaccharides, and gamma-linolenic acid (GLA), necessitates a detailed techno-economic evaluation to ensure commercial viability (Spínola et al. 2024b). Cultivation in controlled photobioreactors (PBRs) is preferred for pharmaceutical-grade biomass due to higher purity and consistency, despite higher capital costs (500,000–2 M/ha) compared to open ponds, which risk contamination (Al-Dailami et al. 2022). Optimal growth conditions (25–35 °C, pH 9–11, and controlled light/nutrients) maximize bioactive yields, with *phycocyanin* production enhanced under specific nitrogen regimes (Soni et al. 2019). Downstream processing, including gentle harvesting (centrifugation or filtration) and freeze-drying, preserves therapeutic compounds but increases costs (50–100/kg), while advanced extraction (chromatography, ultrafiltration) further elevate expenses but yield high-value extracts *(phycocyanin* at 100–500/g) (Gao et al. 2020). The global market for *Spirulina*-derived therapeutics is expanding, particularly in anti-inflammatory, immunomodulatory, and antiviral applications, with clinical validation critical for premium pricing (Htoo et al. 2024). However, compliance with regulatory standards including Good Manufacturing Practices (GMP) and FDA/EMA approvals, coupled with rigorous quality control measures for heavy metals and microbial contaminants, necessitates additional investment but is essential for commercial market penetration. Implementing large-scale production, adopting biorefinery approaches, and utilizing by-product streams (such as converting spent biomass to biofuel feedstocks) collectively enhance both the economic viability and environmental sustainability of operations (Pakdel et al. 2023). For therapeutic applications, the anticipated ROI period generally ranges between five to eight years. The commercial viability can be substantially augmented through strategic collaborations with pharmaceutical partners and the implementation of patent-secured product formulations (Gurgula 2020). This implies that strategic pharma/biotech partnerships and patented formulations emerge as critical factors for enhancing market potential and ensuring competitive advantage. Despite its market value, *Spirulina's* low environmental footprint (CO₂ sequestration, non-arable cultivation) aligns with green medicine trends, though energy-efficient processing remains a challenge. Therefore, investment in research (clinical trials, bioavailability enhancement) and scalable production technologies is key to unlocking full medicinal value of *Spirulina* sustainably.

The environmental applications of Spirulina present a compelling circular economy model where its cultivation simultaneously addresses wastewater treatment, carbon sequestration, and biomass production (González Fernández et al. 2025; Wen et al. 2022). The techno-economic analysis reveals that integrating Spirulina systems with industrial effluents or municipal wastewater can significantly reduce nutrient input costs while achieving 70–90% removal of nitrogen, phosphorus, and heavy metals (Menger-Krug et al. 2012), with biomass productivity of 10–15 g/m^2^/day in such modified systems. The carbon capture potential (1.8 kg CO₂ per kg biomass) creates opportunities for carbon credit generation, though this requires robust monitoring and verification systems (Gao et al. 2020). Capital costs for environmental applications are 20–30% lower than standard cultivation due to the utilization of existing water infrastructure, with operational savings from reduced fertilizer requirements. The produced biomass, while potentially containing contaminants from wastewater, can be safely utilized for bioenergy production (biogas yield of 350–450 mL CH₄/g VS) or as organic fertilizer after proper treatment (Shahid et al. 2020). Economic viability hinges on location-specific factors including wastewater availability, climate conditions, and regulatory frameworks for water reuse and carbon trading (Shahbaz et al. 2025). The payback period for such integrated systems typically ranges from 4–7 years, with dual revenue streams from environmental services (waste treatment fees, carbon credits) and biomass valorization (Saravanan et al. 2023). However, challenges include public acceptance of wastewater-grown biomass and the need for standardized protocols for contaminant testing. Future prospects involve developing modular, scalable systems for decentralized wastewater treatment and carbon mitigation, particularly in industrial zones and rapidly urbanizing areas, with potential synergies with flue gas CO₂ capture from power plants or cement factories.

The potential of *Spirulina* as a bioenergy feedstock is evaluated through its biomass productivity (10–20 g/m^2^/day in open ponds) and biochemical composition, with 5–15% lipids for biodiesel, 15–25% carbohydrates for bioethanol, and 50–70% proteins for biogas via anaerobic digestion (yielding 350–450 mL CH₄/g volatile solids) (Saravanan et al. 2023; Sumprasit et al. 2017b). The production cost of Spirulina biomass (5–12/kg in open ponds) remain a key challenge for bioenergy viability, as current algal-biodiesel costs ($2–8/L) are non-competitive with fossil fuels without subsidies (Lopes et al. 2023). Thus, scale-up economics remain a key challenge for bioenergy viability, as current algal biodiesel production costs ($2–8/L) significantly exceed petroleum diesel benchmarks, necessitating breakthroughs in cultivation productivity, lipid yields, and downstream processing efficiency to achieve commercial competitiveness. However, integrated biorefinery approaches that co-produce high-value compounds (phycocyanin, proteins) alongside biofuels improve economics, with energy returns on investment (EROI) increasing from < 1 (standalone biofuels) to 2–4 when coupled with nutraceutical extraction. Hydrothermal liquefaction (HTL) shows promise for direct conversion of wet biomass into biocrude (yields up to 40%), bypassing energy-intensive drying, while biochemical routes (enzymatic hydrolysis/fermentation) achieve 60–70% carbohydrate-to-ethanol conversion (Li et al. 2022). Capital costs for 10-ha bioenergy-focused facilities range $1.5–3 million, with 7–10-year payback periods contingent on policy support (carbon credits, renewable fuel mandates) and technological advances in strain productivity (genetically enhanced lipid/carbohydrate strains) and harvesting efficiency (low-energy electrocoagulation) (Li et al. 2022). Interestingly, sustainability metrics favor low land-use footprint of Spirulina (100 time less than soy biodiesel) and wastewater remediation potential, though energy-positive operations require solar drying or waste heat integration. However, future viability depends on scaling innovations and policy frameworks that value its environmental co-benefits alongside fuel production.

In the general overview, the feasibility of any *Spirulina* production project depends on a rigorous evaluation of the biomass resource, efficient logistics, and a comprehensive assessment of potential environmental impacts. A critical component of this evaluation is a thorough analysis of the advantages and disadvantages of *Spirulina* production, accounting for environmental uncertainties, ideally through a life cycle assessment (LCA) framework. LCA provides a standardized methodology for quantifying and evaluating the environmental burdens associated with resource utilization, energy consumption, material inputs, and waste generation throughout the entire lifecycle of a product, process, or activity.

The lifecycle of *Spirulina* and its derived bioproducts, from initial cultivation to final disposal or repurposing, comprises a series of interconnected stages that collectively define their environmental and societal impact. This lifecycle begins with the cultivation and harvesting of the *Spirulina* biomass. Subsequently, this biomass undergoes various conversion processes, enabling its application in dietary, medicinal, energy, and environmental remediation contexts. These processed *Spirulina* products then enter the utilization phase, where they are consumed (e.g., as nutritional supplements, pharmaceuticals) or employed in diverse applications (e.g., biofuel production, wastewater treatment). During this utilization phase, the environmental and social performance of the *Spirulina*-based products is monitored and evaluated, considering factors such as greenhouse gas emissions, resource consumption, and economic benefits. Finally, at the end of their functional life, *Spirulina* and its associated products are either recycled, repurposed, or disposed of in an environmentally responsible manner. This holistic, cradle-to-grave LCA approach is essential for understanding the broader implications of these renewable alternatives and for making informed decisions that promote sustainability.

While *Spirulina* presents a promising sustainable resource, it is crucial to recognize that, like other bio-based systems, its production often involves energy-intensive processes. Analogous to the agricultural systems discussed by some researchers (Harun et al. 2021; Jeswani et al. 2020). *Spirulina* cultivation can require significant energy inputs for processes such as water circulation, artificial lighting (where applicable), and temperature regulation. A comprehensive LCA must therefore carefully assess the potential environmental impacts associated with these energy demands, as well as the environmental burdens related to resource acquisition and processing. Similar to the significant contribution of the agricultural phase to the environmental footprint of sugarcane-based bioenergy (Ayodele et al. 2020; Khatri and Pandit 2022), the cultivation and processing stages of *Spirulina* could also represent potential environmental hotspots. However, mirroring the potential of bagasse in sugarcane biorefineries, *Spirulina* itself represents a valuable and potentially sustainable biomass resource applicable to a range of industries. Direct comparisons of the environmental performance of *Spirulina*-derived products with conventional alternatives, following the example of Tsiropoulos et al., in their analysis of Indian and Brazilian bioethanol, are essential for establishing the true sustainability credentials of *Spirulina* (Tsiropoulos et al. 2014). Critically, as emphasized previously, distinguishing between cradle-to-gate and cradle-to-grave LCA methodologies is vital for *Spirulina* to ensure a complete and accurate understanding of its overall environmental impact. Consequently, a rigorous, full lifecycle LCA of *Spirulina*, encompassing all stages from initial cultivation to end-of-life management, is indispensable for realizing its full potential as a truly sustainable resource across diverse applications.

On water and energy demand for the *Spirulina* biomass production, operational costs within the production system, particularly for *Spirulina*, are significantly influenced by water consumption, energy expenditure, and effluent treatment (Acién et al. 2017). Water costs are determined by both water source (seawater, brackish water, freshwater, or wastewater) and the volume required. Furthermore, the costs associated with treating wastewater effluent within the wastewater facility must be factored into the overall cost assessment (Acién et al. 2017).

In a *Spirulina* production, water costs can be mitigated through water recirculation after biomass recovery. Recirculating the culture medium, encompassing both water and nutrients (Acién et al. 2017; de Morais et al. 2015), has been identified as a key strategy for reducing biomass production expenses. Moreover, when the cultivated biomass is intended for energy applications, such as biofuel production, wastewater can be utilized directly in *Spirulina* cultivation (Giwa et al. 2018; Salla et al. 2016; Zhai et al. 2017).

Energy consumption is a critical consideration throughout all stages of biomass production, largely dependent on the specific equipment employed. Energy is required during microalgal cultivation (for reactor operation, culture medium supplementation, and process control), as well as during subsequent stages, including biomass recovery, drying, and fractionation, all necessary to obtain the final bioproduct (Acién et al. 2017).

In another context, considering energy expenditure across cultivation (raceway and culture medium recirculation), biomass recovery (filtration), drying (spray drying), and fractionation, the drying and fractionation stages represent the most substantial energy consumers. While comparing energy costs between spray drying and drum drying, drum drying exhibits lower energy consumption (0.9 kWh kg − 1 evaporated water) than spray drying (1.09 kWh kg − 1 evaporated water). However, the higher drying capacity of spray dryers makes them preferable for large-scale *Spirulina* production systems (Fasaei et al. 2018). A case study by Acién et al., indicates that utility consumption (water, energy, and other) accounts for approximately 5.7% of the total biomass production costs in a raceway bioreactor (600 t year^−1^). Further analysis, separating utility consumption from other biorefinery processes, reveals that energy consumption (reactor operation and biomass recovery) represents 96.2% of the costs, while water consumption, despite evaporative losses (30,000 m^3^ ha^−1^ year^−1^), constitutes only 0.5% of the total costs (Acién et al. 2014).

The techno-economic (TEA) and LCA entails both the economic viability and environmental impacts of the production and usage of *Spirulina*. For their economic viability, raceway open ponds are the main cultivation methods of producing *Spirulina* for commercial use. One of the most popular reactors, raceway reactors, is estimated to cost about $750,000 to construct a 100,000 m^2^ sized reactor (Costa et al. 2019a). Due to its high temperature requirements and need for extended exposure to natural light, commercial production of the *Spirulina* in open ponds has only been possible in tropical and subtropical regions. Open culture systems are widespread due to available sunlight and ease in maintenance. It is true that the widespread use of open culture systems for *Spirulina* production, often attributed to available sunlight and ease of maintenance, highlights a key factor driving their popularity. However, while sunlight is indeed crucial for *Spirulina*'s photoautotrophic growth, converting carbon dioxide and water into biomass using light energy, the relationship between available sunlight and *Spirulina* production is more complex than it initially appears. Sunlight provides the essential energy for photosynthesis, directly influencing photosynthetic rate and biomass productivity, and its utilization eliminates the need for costly artificial lighting, a major economic advantage. The abundance of sunlight in tropical and subtropical regions explains the widespread use of open systems in these areas. However, several challenges and critical considerations arise. Excessively high light intensities can lead to photoinhibition, damaging the photosynthetic machinery and hindering growth, a significant concern in sunny climates where surface cells are exposed to damaging light levels. Light penetration and self-shading within dense cultures limit light utilization efficiency, necessitating mixing to mitigate this. Variations in sunlight's spectral quality, due to atmospheric conditions or time of day, can affect photosynthetic efficiency, suggesting that optimizing the light spectrum could enhance productivity. Seasonal and diurnal variations in sunlight intensity further complicate matters, impacting growth rates and requiring careful planning. Photo acclimation, while allowing *Spirulina* to adjust to varying light intensities, is a process that requires time and energy, and rapid fluctuations can disrupt growth. Finally, the effect of sunlight is intertwined with other environmental factors like temperature, nutrient availability, and CO_2_ concentration. Therefore, optimizing sunlight utilization requires strategies like culture density management to balance light availability and self-shading, effective mixing and circulation to distribute light and nutrients, careful pond design and orientation, and potential research into enhancing light-harvesting pigments. While open ponds rely on natural sunlight, photobioreactors offer greater control, though at a higher cost. In short, harnessing sunlight effectively requires an in-depth understanding of its complex interaction with *Spirulina* growth and implementing appropriate management approaches. Culture medium and harvesting stage constitute 15–25% and 20–30% of total production costs, respectively. The conventional Zarrouk medium which costs about $0.08 per litre is replaced by an alternative medium of similar condition, costing about five times less. Different filtration methods can be used in harvesting the *Spirulina* from reactors, depending on scale and other operational factors. However, such costs are dramatically reduced to as low as $1 to $1.5/m^2^ in raceway ponds (Costa et al. 2019b). Drying is prominently performed with spray dryer and drum dryer, each with its pros and cons. The drum dryer has lower energy cost compared to the spray dryer while it costs higher to purchase and maintain. With the above cost of production, a critical analysis of *Spirulina* production costs reveals that the culture medium, harvesting, and drying stages represent substantial portions of the overall expenditure. The culture medium, providing essential nutrients, can be a significant cost driver, with the conventional Zarrouk medium costing around $0.08 per litre. However, alternative media, costing five times less (approximately $0.016 per litre), offer a significant economic advantage, likely due to the use of less refined or locally sourced ingredients, potentially including agricultural by-products or industrial waste streams. Yet, simply reducing cost isn't sufficient; the alternative medium must maintain nutrient balance, purity and consistency, scalability, and long-term stability. Therefore, while the potential cost reduction is attractive, thorough evaluation of the alternative medium's performance is critical, and R&D focused on optimizing low-cost media using local resources is crucial. Harvesting, separating *Spirulina* biomass, is another major cost driver. Costa et al. (2019b) indicate harvesting costs can be reduced to $1-$1.5/m^2^ in raceway ponds, suggesting simple filtration techniques like fabric or mesh filters or flotation. However, biomass recovery efficiency, water reuse, and compatibility with post-harvest processing must be considered. Finally, drying, crucial for preservation, often involves spray drying (energy-intensive) or drum drying (higher capital/maintenance costs). The choice depends on production scale and desired product form, and research into more energy-efficient methods is needed. Overall cost optimization requires a holistic approach, considering the interplay between all stages. A comprehensive cost analysis is crucial, and exploring integration with other processes like wastewater treatment can offer further cost reduction and resource recovery opportunities.

Open pond raceway systems are the dominant method for commercial *Spirulina* production, largely due to their perceived economic viability, though a closer examination reveals a complex interplay of factors influencing their true cost-effectiveness and sustainability. While the initial capital expenditure for raceway construction, estimated at $750,000 for a 100,000 m^2^ system (Costa et al. 2019a), might seem substantial, it's crucial to dissect the operational costs and inherent limitations of this approach. Open ponds offer the key advantage of readily available sunlight, a crucial requirement for *Spirulina* growth, reducing the need for artificial lighting and associated energy costs, particularly beneficial in tropical and subtropical regions. They are also often perceived as simpler to operate and maintain compared to closed systems like photobioreactors, with tasks like mixing, nutrient addition, and harvesting being less technically demanding. However, this apparent simplicity is countered by significant challenges. The reliance on natural sunlight and specific temperature requirements restricts commercial *Spirulina* production in open ponds to tropical and subtropical regions, limiting wider global production and potentially increasing transportation costs. Open systems are inherently susceptible to contamination from various sources, including microbial contaminants (bacteria, fungi, and other *Spirulina* that can outcompete or predate on *Spirulina*), environmental contaminants (dust, airborne particles, and pollutants), and grazing zooplankton, all of which can reduce yields and product quality. Maintaining optimal temperature in open ponds can be difficult, especially in regions with fluctuating weather, negatively impacting *Spirulina* growth. Water loss due to evaporation, particularly in hot and dry climates, necessitates substantial water replenishment and can contribute to water scarcity. Nutrient management, while potentially cheaper with alternative media compared to the conventional Zarrouk medium, still constitutes a significant operational cost, ranging from 15 to 25% of total production costs. Optimizing nutrient supply is a complex task. Harvesting, comprising 20–30% of total production costs, involves separating the *Spirulina* biomass, and while Costa et al., suggest lower costs ($1-$1.5/m^2^) for filtration, the specific methods and their efficiency greatly influence this cost (Costa et al. 2019a). The subsequent drying process, whether using spray dryers or drum dryers, adds further expense, with trade-offs between energy consumption and capital/maintenance costs. Open ponds often exhibit lower productivity compared to closed photobioreactors, and scaling up production can be challenging due to the increasing difficulty in managing environmental factors and contamination risks. Finally, while *Spirulina* itself can be sustainable, the environmental footprint of open pond cultivation, including water consumption, energy use, nutrient inputs, and potential waste generation, requires careful assessment. Therefore, future development should focus on optimizing pond design and operation, developing cost-effective media and nutrient management strategies, improving harvesting and drying technologies, and exploring integration with wastewater treatment or other systems. Crucially, conducting a comprehensive Life Cycle Assessment is essential for understanding the true environmental impact and identifying areas for improvement.

About 7.7 kg of CO_2_ is emitted for every 1 kg of *Spirulina* tablet produced. Most of this comes from bicarbonate (35.4%) and electricity (21.4) during cultivation (82%) (Ye et al. 2018). The environmental footprint from chemicals and nutrients contained in the medium is also reported to constitute a large part of the high environmental impacts in the cultivation stage. Other molecules, such as O_3_, SO_2_, and N were released either in the atmosphere or water. The harvest stage has less impact on the environment compared to the cultivation stage, which can be further reduced by treating the biomass residues. Reports on different microSpirulina report electricity usage from drying and dewatering of the harvesting stage to have higher environmental impacts in biofuel production (Ye et al. 2018). Despite the discord about which stage or the order of factors for high environmental impacts, medium, electricity, bicarbonate, urea, and inorganic carbonates are agreed to be the cause. Ecotoxicity study implied acute toxicity at 69.9 CTUe, largely due to bicarbonate and buffer. Such emissions are estimated to be 2 to 5 times higher in making food products from *Spirulina* than biofuels (Ye et al. 2018). *Spirulina* cultivation can be resource-efficient, especially when integrated with renewable energy sources. For instance, a study on large-scale *Spirulina* production in Iceland's Hellisheidi geothermal park demonstrated significantly lower environmental impacts compared to conventional livestock farming. Replacing 1 kg of beef with 1 kg of *Spirulina* could save approximately 100 kg of CO₂-equivalent greenhouse gases (Tzachor et al. 2022). *Spirulina* cultivation can help combat pollution, water contamination, and overconsumption, contributing to environmental sustainability (Tzachor et al. 2022).

To promote the sustainability and economic viability of the *Spirulina*, it is paramount to promote practices that will improve the production and environmental performance of its biomass such as integrating circular economy principles (e.g., using agricultural residues as nutrients). Environmental effects of producing 1 kg of *Spirulina* tablet include global warming, smog, eutrophication, acidification, and fossil fuel production. Addressing environmental bottlenecks such as reducing energy consumption, waste, and emissions, optimizing culture conditions, and using renewable energy and mineral-rich water will boost production of *Spirulina*. Integrated systems are often used to cultivate the *Spirulina* for commercial production, where they utilize industrial or municipal waste to supply water, nitrogen, phosphorous, organic carbon, and nutrients. This practice can reduce emission of greenhouse gases, cost of wastewater treatment, and environmental impacts, while providing nutrients to the *Spirulina*. Additionally, using wastewater coupled with recycling water and nutrients after biomass recovery is a suggested approach to cut the cost of producing biomass in biorefineries (Costa et al. 2019a). Elaborating on circular practices in *Spirulina* production, specifically nutrient recycling and waste valorisation, reveals a growing focus on sustainability. Nutrient recycling strategies include utilizing digestate from anaerobic digestion of organic wastes as a cost-effective nutrient source, closing the loop on nutrient flow, with ongoing research optimizing digestate composition and application. Nutrient recovery from wastewater, often rich in nitrogen and phosphorus, involves treating wastewater for *Spirulina* growth and then recovering remaining nutrients for reuse, minimizing nutrient loss. Even *Spirulina* processing waste streams can be a source of recyclable nutrients through composting or anaerobic digestion. Waste Valorization practices encompass using agricultural residues, like crop waste or animal manure, processed into nutrient-rich substrates for *Spirulina* growth. Certain industrial waste streams, after careful analysis and treatment, can supplement nutrients, providing trace elements or minerals. *Spirulina* cultivation itself contributes to CO_2_ sequestration, utilizing industrial emissions, reducing greenhouse gases, and providing a carbon source. The production and utilization approach integrates various processes to utilize all biomass components and waste streams, extracting valuable compounds, using residual biomass for biofuel, and recovering nutrients for recycling. However, challenges remain, including ensuring nutrient availability and bioavailability from recycled materials, removing potential contaminants from waste streams, achieving scalability and consistency in waste processing, and demonstrating the economic viability of these practices.

The economic viability of *Spirulina* production is influenced by factors such as cultivation methods, energy consumption, and market demand. A techno-economic analysis indicated that a *Spirulina* powder production plant with an 80 m^3^ photobioreactor capacity could be profitable, achieving a payback period of approximately 6.34 years under specific operating conditions. *Spirulina* production has the potential to innovate the agri-food industry and generate much-needed income and jobs in various regions. For example, a feasibility study in Morocco suggested that *Spirulina* cultivation could increase sustainability by enhancing resilience to climate change and reducing reliance on protein imports (Rahmann et al. 2021). While *Spirulina* cultivation can be resource-efficient, energy consumption remains a critical factor. Optimizing energy use in cultivation and processing is essential to enhance economic feasibility and minimize environmental impacts. The initial capital investment and ongoing operational expenses, including energy, labour, and nutrient inputs, can be substantial. Developing cost-effective cultivation and harvesting techniques is crucial to improve economic viability (Costa et al. 2019a). *Spirulina* has diverse applications across various industries. Table 6 contained comparative summary on the advantages and limitations of *Spirulina* for different applications.

**Table 6:** Summary of the advantages and limitations of *Spirulina* in various applications

Application	Advantage	Limitation
Nutritional Supplement	- High protein content (55–70% dry weight), surpassing traditional protein sources like beef and soybeans (Podgórska-Kryszczuk 2024)	- Potential contamination with heavy metals or toxins if not properly cultivated (AlFadhly et al. 2022a, b)
	- Rich in essential amino acids, vitamins, and minerals (Costa et al. 2019a)	- May cause allergic reactions in sensitive individuals (AlFadhly et al. 2022a, b)
	- Contains antioxidants with potential health benefits (A. Kumar et al. 2022a, b)	- Limited vitamin B12 bioavailability (AlFadhly et al. 2022a, b)
Biofuel Production	- Rapid growth rate and high biomass yield (Podgórska-Kryszczuk 2024)	- Relatively low lipid content, making biodiesel production less efficient (Podgórska-Kryszczuk 2024)
	- Can be cultivated on non-arable land, not competing with food crops (Podgórska-Kryszczuk 2024)	- Requires optimization of cultivation and processing methods to enhance biofuel yields (Podgórska-Kryszczuk 2024)
	- Potential for bioethanol and biogas production (Podgórska-Kryszczuk 2024)	
Cosmetics and Pharmaceuticals	Contains bioactive compounds with antioxidant, anti-inflammatory, and antimicrobial properties (Bortolini et al. 2022)	Stability and efficacy of active compounds can be affected by processing and formulation (Bortolini et al. 2022)
	Potential use in anti-aging and skin care products (Bortolini et al. 2022)	Regulatory challenges in standardizing extracts for pharmaceutical use (Bortolini et al. 2022)
Food Colorant	Natural pigment source (phycocyanin) offering an alternative to synthetic colorants (Calovi and Rossi 2023)	Sensitivity to light and heat may affect colour stability (Calovi and Rossi 2023)
	Biodegradable and environmentally friendly (Calovi and Rossi 2023)	Potential for batch-to-batch variability in pigment concentration (Calovi and Rossi 2023)

On the market feasibility of the main *Spirulina* products, the high nutritional value of *Spirulina* biomass has fueled market growth to meet increasing consumer demand. This microalga is primarily sold in products for human consumption, including food supplements (powders, capsules, and tablets) and animal feed. *Spirulina* is also incorporated as a functional ingredient in various food products, such as juices, desserts, cakes, pasta, salads, cookies, breakfast cereals, snacks, instant soups, cereal bars, beer, tea, chocolate, honey, and other beverages (Luo et al. 2024; Wu et al. 2023). Large-scale *Spirulina* production began in the 1970s at Lake Texcoco, Mexico, where natural blooms were harvested and commercialized until 1995 (AlFadhly et al. 2022a, b). In 1978, Dainippon Ink & Chemicals Inc. (DIC) of Thailand pioneered commercial-scale *Spirulina* cultivation in artificial ponds (Costa et al. 2019a).

Over the past four decades, the market has expanded, with numerous companies specializing in large-scale *Spirulina* production. The Food and Agriculture Organization of the United Nations (FAO) reports *Spirulina* production in at least 22 countries, spanning Africa, the Americas, Asia, and Europe. North American and Asia–Pacific production contributes approximately 2000 t year^−1^ (FAO., 2022). Chinese production is estimated to reach approximately 10,000 t year^−1^, based on company data, with other estimates exceeding 8500 t year^−1^ (Costa et al. 2019a). The key market players include Cyanotech Corporation (USA), Parry Nutraceuticals (India), Fuqing King Dirmsa *Spirulina* Co., Ltd. (China), *Spirulina* Mater © (Chile), Dongtai City *Spirulina* Bio-engineering Co., Ltd (China), Boonsom *Spirulina* Farm (Thailand), Olson Nutrition Ltd. (Brazil), Fazenda Tamanduá (Brazil), and Vital Brazil (Brazil). Earthrise Nutritionals, LLC (USA) and Hainan-DIC MicroSpirulina (China), both subsidiaries of DIC Corporation, produce approximately 550 and 350 t year^−1^, respectively (Costa et al. 2019a). These companies primarily market *Spirulina* biomass in powder, capsule, and tablet forms, as well as phycocyanin pigment. Earthrise Nutritionals is currently the world's largest *Spirulina* producer, making DIC Corporation the largest global entity in *Spirulina* production when combined with Hainan-DIC MicroSpirulina's output (Costa et al. 2019a).

### Limitations and future perspectives

The *Spirulina* possess diverse therapeutic properties due to its rich composition of bioactive compounds. While the effects of some constituent compounds such as phyocyanin, chlorophyll, carotene, xanthophylls, flavonoids, vitamins, minerals etc., have been extensively studied there is still inadequate reports on specific mechanisms of action for all its therapeutic activities. Hence, there is need for more pre-clinical and clinical studies on known therapeutic activities of *Spirulina* along with toxicological studies. Its potential effects need to be also explored more in neurodegenerative diseases and respiratory diseases. This is to further facilitate its application in the pharmaceutical and nutraceutical industries.

Extreme environmental requirements and cost of production are the major bottlenecks that must be overcome to achieve sustainable commercial cultivation of *Spirulina*. Open pond cultivation is the widespread method used in commercial production of *Spirulina* due to its relative ease and low cost. However, due to the requirement for high temperature, consistent sunlight, and alkaline pH conditions, nations outside tropical and subtropical regions are unable to cultivate *Spirulina* in open ponds thereby relying on closed photobioreactors. Depending on size, a raceway bioreactor costs about $500,000 (50,000 m2) to $725,000 (100,000) which makes production less feasible in underdeveloped countries. Furthermore, pond cultivation of *Spirulina* remains at great risk for contamination by diverse microzooplanktons that can affect yield and cause losses (Yuan et al. 2017).

Genetic and RNA studies can provide better understanding of metabolic pathways in *Spirulina* to allow for better modification of its strains that for ease of cultivation and processing. Genetic tools were previously used to express therapeutic proteins in *Spirulina* exogenously. The strain has successfully scaled through phase 1 of clinical trial in oral antibody delivery in mice, for disease prevention (Jester et al. 2022). Challenges in productivity, sustainability, and resilience have hitherto been successfully addressed in aquaculture, crop, and livestock production using genetic tools. *Spirulina* production can be globally upscaled and improved with genetic modifications that enhance resilience, increase yield, and reduce environmental impact.

### Roadmap for future research

*Spirulina* holds significant promise across various sectors, necessitating a comprehensive research roadmap to fully harness its potential. Key areas for future investigation include optimizing cultivation methods to enhance biomass yield and bioactive compound production, exploring genetic and metabolic engineering to improve desired traits, and developing sustainable harvesting and processing techniques to reduce costs and environmental impact. Additionally, rigorous clinical studies are essential to validate *Spirulina*'s therapeutic applications, particularly its antioxidant, anti-inflammatory, and anticancer properties. Research into its environmental applications, such as wastewater treatment and carbon sequestration, could further contribute to sustainability efforts. Understanding market dynamics and consumer acceptance will facilitate the development of *Spirulina*-based products, while exploring its role in space biotechnology could support life in extra-terrestrial environments. By addressing these areas, future research can unlock the full potential of *Spirulina* in health, sustainability, and technology.

On the other hands, deeper into the core problems and proposed solutions across various fields:

### High production costs & environmental dependence

i. **Elaboration:** *Spirulina*'s commercial viability is hampered by the high costs associated with cultivation, particularly the energy demands for maintaining optimal temperature and light, as well as the cost of nutrient media. Open pond systems, while cheaper, are heavily reliant on specific climatic conditions, limiting production to tropical and subtropical regions. Closed photobioreactors, though offering better control, are significantly more expensive, putting them out of reach for many potential producers, especially in developing nations. This dependence on specific environments also creates sustainability concerns related to resource consumption and potential pollution.
ii. **Proposed Solutions:**
  - **Cost-Effective Nutrient Media:** Research and development into cheaper, locally sourced, and sustainable nutrient media are crucial. This includes exploring agricultural waste, wastewater, and other readily available resources as nutrient sources.
  - **Energy Efficiency:** Implementing energy-efficient technologies in both open pond and closed photobioreactor systems is essential. This can involve optimizing pond design for light capture, utilizing renewable energy sources (solar, wind), and improving temperature control mechanisms.
  - **Genetic Improvement:** As we mentioned, genetic modification holds immense potential. Developing *Spirulina* strains with enhanced growth rates, improved nutrient utilization, increased tolerance to environmental stress (temperature, salinity), and resistance to contamination can significantly reduce production costs and expand cultivation possibilities. This is in response to acknowledged gap between research and commercialization. Because while genetic modification (GM) of *Spirulina* has shown promising results in laboratory settings, particularly for enhancing lipid accumulation (20–30% increases in some studies) and carbohydrate content for biofuel production, a significant barriers impede its transition to commercial-scale applications [29]. Thus, while genetic modification has demonstrated potential to enhance bioenergy traits of Spirulina (e.g., lipid productivity), its application remains experimental due to regulatory and scalability challenges. However, near-term bioenergy gains may instead rely on process engineering and non-GM strain selection.
  - **Integrated Systems:** Combining *Spirulina* cultivation with other systems, such as wastewater treatment or biogas production, can create synergistic benefits. Wastewater can provide nutrients, and biogas production can generate energy, reducing overall costs and environmental impact.

### Contamination and yield variability

i. **Elaboration:** Open Pond systems are highly susceptible to contamination by bacteria, fungi, other *Spirulina*, and zooplankton. Contamination can drastically reduce yields, compromise product quality, and even lead to complete crop loss. This variability in yield makes large-scale, consistent production challenging.
ii. **Proposed solutions**
  - **Improved pond management:** Implementing better pond management practices, including regular cleaning, filtration, and monitoring for contaminants, can help minimize contamination risks.
  - **Biological control:** Exploring the use of beneficial microorganisms or other biological control agents to suppress the growth of contaminating organisms could offer a more sustainable approach.
  - **Closed systems (PBRs):** While more expensive, closed photobioreactors offer a more controlled environment, significantly reducing the risk of contamination. Research is ongoing to make PBRs more economically viable.
  - **Genetic engineering:** Developing *Spirulina* strains with enhanced resistance to specific contaminants can provide a more robust and reliable production system.

### Limited understanding of therapeutic mechanisms & applications

i. **Elaboration:** While *Spirulina* is recognized for its nutritional and therapeutic potential, the specific mechanisms of action for many of its health benefits are not fully understood. This lack of understanding limits its wider application in the pharmaceutical and nutraceutical industries. Furthermore, its potential in treating various diseases, particularly neurodegenerative and respiratory diseases, requires further investigation.
ii. **Proposed solutions**
  - **Advanced research:** More pre-clinical and clinical studies are needed to elucidate the mechanisms by which *Spirulina*'s bioactive compounds exert their therapeutic effects. This includes investigating its potential in treating specific diseases.
  - **Standardization and quality control:** Developing standardized methods for *Spirulina* cultivation, processing, and quality control is crucial for ensuring consistent product quality and efficacy. This will build consumer trust and facilitate its wider adoption.
  - **Product development:** Further research and development are needed to create innovative *Spirulina*-based products for various applications, including functional foods, dietary supplements, and pharmaceuticals.

### Scaling up production & processing

i. **Elaboration:** Meeting the growing global demand for *Spirulina* requires scaling up production and processing capabilities. This involves not only increasing the size of cultivation systems but also optimizing harvesting, drying, and other downstream processing steps.
ii. **Proposed solutions**
  - **Automation & process optimization:** Implementing automation and optimizing various processing steps can improve efficiency and reduce costs associated with large-scale production.
  - **Biorefinery approach:** As mentioned before, the biorefinery concept offers a sustainable and efficient way to utilize all components of the *Spirulina* biomass, maximizing product recovery and minimizing waste.
  - **Technological advancements:** Continued research and development in areas like cultivation technology, harvesting methods, and drying techniques are essential for achieving cost-effective and sustainable large-scale production.

## Conclusion

This review has synthesized the diverse potential of *Spirulina* across medicinal, environmental, and energy-based applications, highlighting its remarkable versatility and promising future. Our recap of key findings underscores *Spirulina*'s rich nutritional profile, its demonstrated therapeutic properties attributed to a complex array of bioactive compounds, and its potential for bioremediation and bioenergy production. From its established role as a valuable food supplement to its emerging applications in pharmaceuticals, wastewater treatment, and biofuel production, *Spirulina* presents a compelling case for its broader integration into various sectors.

Harnessing the full power of *Spirulina* requires a concerted and interdisciplinary effort. While significant progress has been made in understanding its nutritional and therapeutic benefits, further research is crucial to fully elucidate the specific mechanisms of action for its diverse therapeutic activities. This includes comprehensive pre-clinical and clinical trials, coupled with toxicological studies, to solidify its position in the pharmaceutical and nutraceutical industries. Furthermore, exploring *Spirulina*'s potential in addressing critical health challenges, such as neurodegenerative and respiratory diseases, warrants further investigation.

Beyond its medicinal applications, *Spirulina* offers significant promise for addressing pressing environmental concerns. Its ability to remediate wastewater and soil, sequester CO_2_, and contribute to biofuel production positions it as a key player in the transition towards a sustainable bioeconomy. In fact, the *Spirulina* exhibited exceptional biosorption capabilities for uptake of heavy metals, dyes and other inorganic and organic contaminants from soil and water environment, making it valuable tool for wastewater treatment and soil remediation. However, realizing this potential requires overcoming critical bottlenecks, particularly the high production costs and environmental sensitivities associated with large-scale cultivation. Addressing these challenges necessitates innovative solutions, including the development of cost-effective and sustainable nutrient media, optimization of cultivation systems for enhanced energy efficiency and reduced contamination risks, and the integration of circular economy principles, such as nutrient recycling and waste valorization.

While the techno-economic and life cycle assessments aspects demonstrated commercial viability of the *Spirulina* for the circular economy applications, further research is imperative to optimize its cultivation, enhance its production yields, and address its potential limitations. Moreover, large-scale production and market penetration strategies are crucial for realizing the full potential of *Spirulina* as sustainable and multifunctional bioresource.

A call for interdisciplinary research and collaboration is therefore paramount. Bridging the gap between science and engineering, surface chemistry, plant biology and genetics, and wastewater treatment engineering and technology is essential to translate the promising potential of *Spirulina* into practical, real-world applications. This collaborative approach will raise innovation in areas such as strain improvement through genetic engineering, development of novel bioreactor designs, optimization of downstream processing techniques, and the creation of integrated biorefinery and bioeconomic systems. By promotion of communication and knowledge exchange among these diverse disciplines, the scientists and engineers can accelerate the development of sustainable and economically viable *Spirulina*-based solutions that address critical challenges in human health, environmental sustainability, and energy security. Ultimately, this collaborative endeavor will pave the way for unlocking its fullest potential and realizing the multifaceted importance of *Spirulina* as a valuable resource for a healthier planet and a more sustainable future.

## Data Availability

No datasets were generated or analysed during the current study.
