# Correction: A review on techno-economic assessment of *Spirulina* for sustainable nutraceutical, medicinal, environmental, and bioenergy applications

**DOI:** 10.1186/s40643-026-01053-0

**Published:** 2026-04-22

**Authors:** Musa Nasiru Musa, Ghazali Musa Jirgi, Zakariyya Uba Zango, Mannawi Nasiru Isa, Muhammad Abdurrazak, Adamu Ahmad Adamu, Ismael A. Wadi, Adekunle Akanni Adeleke, Zaharaddeen N. Garba, Usman Bello, Haruna Adamu, Ahmad Hosseini-Bandegharaei, Dmitry Olegovich Bokov

**Affiliations:** 1 Department of Biochemistry, College of Natural and Applied Science, Al-Qalam University Katsina, Katsina, 2137 Nigeria; 2 Institute of Semi‑Arid Zone Studies, Al-Qalam University Katsina, Katsina, 2137 Nigeria; 3 Department of Chemistry, College of Natural and Applied Science, Al-Qalam University Katsina, Katsina, 2137 Nigeria; 4 Department of Physics, College of Natural and Applied Science, Al-Qalam University Katsina, Katsina, 2137 Nigeria; 5 Department of Biochemistry, School of Science and Information Technology, Skyline University Nigeria, Zaria Road Kano, Kano, Nigeria; 6 Department of Physiotherapy, Aminu Kano Teaching Hospital, PMB 3452, Kano, Nigeria; 7 Prince Sattam Bin Abdulaziz University, Basic Science Unit, Alkharj 16278, Alkharj, Saudi Arabia; 8 Department of Mechanical Engineering, Nile University of Nigeria, Abuja, Nigeria; 9 Department of Chemistry, Ahmadu Bello University, Zaria, 810107 Nigeria; 10 Biofuel and Biochemical Research Group, Department of Chemical Engineering, Universiti Teknologi, PETRONAS, 32610 Seri Iskandar, Malaysia; 11 Department of Chemistry, Abubakar Tafawa Balewa University, Gubi Campus, Bauchi, 740102 Nigeria; 12 Department of Environmental Management Technology, Abubakar Tafawa Balewa University, Yelwa Campus, Bauchi, 740272 Nigeria; 13 Faculty of Chemistry, Semnan University, Semnan, Iran; 14 Research and Innovation Cell, Rayat Bahra University, Mohali, Punjab India; 15 Department of Sustainable Engineering, Saveetha School of Engineering, SIMATS, Chennai, Tamil Nadu 602105 India; 16 Institute of Pharmacy Named After A.P. Nelyubin, Sechenov First Moscow State Medical University, 8 Trubetskaya St., Bldg. 2, Moscow, 119991 Russian Federation; 17 Laboratory of Food Chemistry, Federal Research Center of Nutrition, Biotechnology and Food Safety, 2/14 Ustyinsky Pr., Moscow, 109240 Russian Federation

**Correction to: Bioresources and Bioprocessing (2025) 12:51** 10.1186/s40643-025-00888-3

In this article (Musa et al. 2025), the author’s name Mannawi Nasiru Isa was incorrectly written as Marnawi Nasiru Isah. The original article has been corrected.
